# An in vivo platform to select and evolve aggregation-resistant proteins

**DOI:** 10.1038/s41467-020-15667-1

**Published:** 2020-04-14

**Authors:** Jessica S. Ebo, Janet C. Saunders, Paul W. A. Devine, Alice M. Gordon, Amy S. Warwick, Bob Schiffrin, Stacey E. Chin, Elizabeth England, James D. Button, Christopher Lloyd, Nicholas J. Bond, Alison E. Ashcroft, Sheena E. Radford, David C. Lowe, David J. Brockwell

**Affiliations:** 1 Astbury Centre for Structural Molecular Biology, University of Leeds, Leeds, LS2 9JT UK; 2 School of Molecular and Cellular Biology, Faculty of Biological Sciences, University of Leeds, Leeds, LS2 9JT UK; 3 AstraZeneca, Granta Park, Cambridge, CB21 6GH UK; 4 Present Address: AstraZeneca, Granta Park, Cambridge, CB21 6GH UK

**Keywords:** Protein aggregation, Biologics

## Abstract

Protein biopharmaceuticals are highly successful, but their utility is compromised by their propensity to aggregate during manufacture and storage. As aggregation can be triggered by non-native states, whose population is not necessarily related to thermodynamic stability, prediction of poorly-behaving biologics is difficult, and searching for sequences with desired properties is labour-intensive and time-consuming. Here we show that an assay in the periplasm of *E. coli* linking aggregation directly to antibiotic resistance acts as a sensor for the innate (un-accelerated) aggregation of antibody fragments. Using this assay as a directed evolution screen, we demonstrate the generation of aggregation resistant scFv sequences when reformatted as IgGs. This powerful tool can thus screen and evolve ‘manufacturable’ biopharmaceuticals early in industrial development. By comparing the mutational profiles of three different immunoglobulin scaffolds, we show the applicability of this method to investigate protein aggregation mechanisms important to both industrial manufacture and amyloid disease.

## Abstract (web summary)

Protein aggregation remains a significant challenge for manufacturing of protein biopharmaceuticals. Here, the authors demonstrate the use of directed evolution and an assay for in vivo innate protein aggregation-propensity to generate aggregation-resistant scFv fragments.

## Introduction

Over the last 30 years, recombinant antibodies have emerged as highly effective therapeutics^1,2^. Antibody-based medicines now comprise over half of first-time approvals^3^, and seven of the ten highest grossing pharmaceuticals in 2018 were based on antibodies or antibody-like scaffolds^4^. This success, and the development of more sophisticated therapeutic strategies based on antibody scaffolds that incorporate multi-dentate interactions and/or effector functions^5,6^, is partly due to the ability to readily generate high affinity candidate therapeutics using hybridoma or phage display platforms^7–9^. While the structural and biophysical properties of antibodies and other protein scaffolds allow the formation of highly avid complexes, the inherent metastability of proteins can result in local or global unfolding that can lead to inactivation and/or protein aggregation. Here, we define aggregates as all species with higher molecular weight than the soluble monomer. This encompasses stable or transient interactions between ordered or disordered states. Aggregation can also be triggered by native-state interactions (colloidal or hydrophobic). As proteins are subjected to various stresses during manufacturing that increase the risk of protein misfolding and aggregation^10^, overcoming aggregation (which may be associated with low protein stability and/or low solubility) is a major hurdle in the development of biopharmaceuticals. Aggregation compromises the quality, stability, and even safety of a drug product^11–13^, yet our ability to identify ‘manufacturable’ candidates with long-term stability during lead isolation and optimisation remains challenging. Similarly, our ability to predict the in vivo aggregation propensity of intrinsically disordered proteins and globular proteins associated with protein aggregation diseases^14^ and how subtle sequence changes alter aggregation in vivo/in vitro are also currently beyond our means. One reason for this is a lack of structural and molecular understanding of the mechanism(s) of the initiation and propagation of protein aggregation, making development of a suitable screen difficult^11^. For biopharmaceuticals, the relationship between different ‘developability’ assays has recently been delineated^15^. However, the ability of these assays to predict manufacturability and long-term stability remains poor^16^, due to lack of a known key quality attribute for aggregation resistance.

A variety of in silico tools have recently been developed to identify aggregation-prone sequences to guide rational design of proteins with enhanced properties^17–22^, for example, by identifying regions of poor solubility^18,23^ in the primary sequence or three dimensional structure of a protein. While prediction of sequences with high aggregation propensity^19,24^ or low solubility^18,22^ is possible, predicting which of these aggregation-prone regions (APRs) will become exposed (or sequestered) by protein folding or unfolding events remains a significant challenge. Such complexity is highlighted by recent work on Tau, an intrinsically disordered protein whose aggregation is linked to neurodegenerative disease^25,26^. Molecular dynamics simulation is an attractive option to identify such APRs^27^, but the necessity of a structural model, length of computational time, the need for a greater understanding of the conformational fluctuations that trigger aggregation, and the availability of suitable force-fields to replicate the stresses found in manufacturing make this approach challenging.

**Fig. 1:**
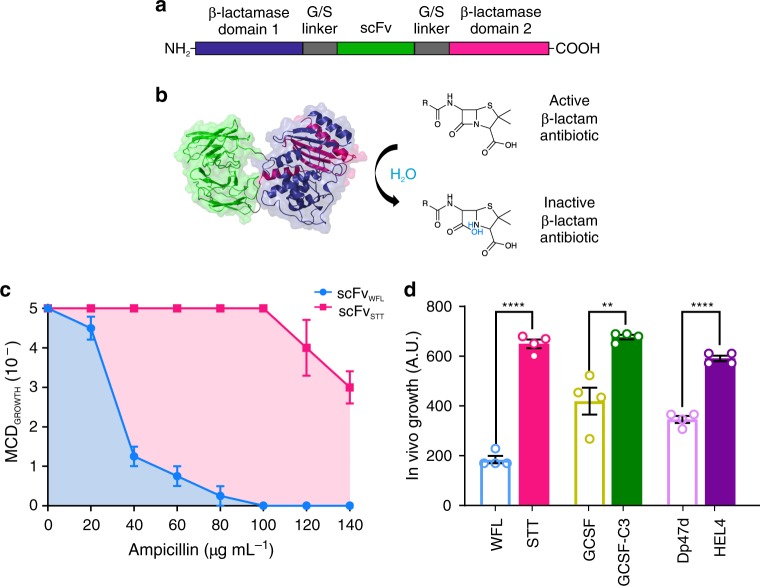
The tripartite β-lactamase assay. **a** The test protein (green) is inserted into a 28-residue glycine/serine-rich linker (grey) separating the two domains of the *E. coli* enzyme TEM-1 β-lactamase (purple and pink). **b** Correct folding of the test protein in the *E. coli* periplasm enables the two halves of β-lactamase to be brought into close proximity to form the functional enzyme active site that hydrolyses β-lactam antibiotics. **c** Antibiotic survival curve of the maximal cell dilution allowing growth (MCD_GROWTH_) on solid medium over a range of ampicillin concentrations for bacteria expressing the aggregation-prone scFv_WFL_ within β-lactamase (blue) or the aggregation-resistant sequence scFv_STT_ (pink). **d** Calculating the area under the antibiotic survival curves (blue and pink shaded area, **c**) yields a single value to compare the behaviour of the different sequences. Data are shown for three aggregation-prone model therapeutic proteins (open bars) and their engineered aggregation-resistant counterparts (solid bars). Data represent mean values ± s.e.m. (*n* = 4 biologically independent experiments). Asterisks denote significance: **p < 0.002, ****p < 0.0001 (two-sided *t*-test). Source data are provided as a Source Data file.

In principle, directed evolution methods using phage, ribosome, or yeast display are powerful approaches capable of investigating the effects of sequence changes on protein aggregation. For example, biopharmaceutical model proteins have been generated, a priori, with enhanced soluble expression^28^, thermodynamic stability^29^ or resistance to heat- or acid-induced aggregation^30,31^. As aggregation can occur by a variety of mechanisms including partial unfolding and homo- or hetero-typic interactions in the native state, no singular property drives aggregation. Consequently, development of a suitable screen to enable the selection and optimisation of biopharmaceuticals for resistance to innate aggregation by directed evolution has not been possible. We have previously developed a tripartite β-lactamase enzyme assay (TPBLA) (Fig. 1a, b) that allows the identification and ranking of aggregation-prone peptides, including the Alzheimer’s peptide (Aβ_40/42_) and islet amyloid polypeptide (IAPP)^32^. In this assay, the test protein is fused in-frame between the two domains of the *E. coli* periplasmic enzyme β-lactamase (βLa, Fig. 1a, b). This assay thus directly links the aggregation-propensity of the test protein to the susceptibility of the bacterium to β-lactam antibiotics^32^. Importantly, by relying solely on the innate aggregation propensity of the protein of interest, the screen does not use arbitrary methods to destabilise proteins (e.g. heat and chemical denaturation^33^) that may not reflect the inherent dynamics of the test protein relevant to aggregation during biomanufacture or in disease^32,34^.

Here, using both therapeutically relevant proteins and proteins involved in aggregation disease as examples, we show that the TPBLA can be used to assess the aggregation propensity of a variety of protein structural scaffolds, including scFv fragments from two monoclonal antibodies (mAbs) that differ by just three amino acids in their V_H_ domains, but which have fundamentally different aggregation properties^35^. We then show that the TPBLA can be used as a screen for directed evolution experiments to select for sequences that are aggregation-resistant. Importantly, the approach does not require any structural knowledge or prior biophysical information about the protein of interest, and can be used to reveal residues that modulate aggregation that could not be predicted a priori using currently available algorithms. At a fundamental level, the ability to detect multiple aggregation liabilities simultaneously enables both spatially clustered and more subtle pair-wise interactions that dictate aggregation to be identified, allowing the delineation of aggregation hotspots in both industrially-relevant and medically-important proteins. In addition, identifying large numbers of sequence variants that endow protection from aggregation will empower the development of algorithms better trained to predict aggregation of biologics and other protein scaffolds. This will allow a greater understanding of the relationship between sequence, solubility and aggregation, the developability of promising biologic candidates and the prediction of mutations that may cause protein aggregation disease.

## Results

### Protein aggregation correlates with bacterial survival

The TPBLA has been used previously to rank the aggregation propensity of intrinsically disordered proteins and two pairs of globular proteins^32^: β_2_microglobulin and D76N (an aggregation-prone natural sequence variant), and Dp47d, a single V_H_ domain nanobody and its non-aggregating counterpart Hel4^30^. To determine whether the assay is able to differentiate between aggregating and non-aggregating sequences of therapeutically relevant protein scaffolds, we compared the in vivo growth scores (area under the antibiotic survival curve, Fig. 1c, d) of Dp47d and its non-aggregation counterpart HEL4, alongside two other aggregation-prone therapeutically relevant protein scaffolds: granulocyte colony-stimulating factor (GCSF) (a 174 residue four helical bundle protein whose poor soluble expression in *E. coli* was improved 1000-fold (GCSF C3) by ribosome display and three parallel selection pressures^36^), and the single chain variant (scFv) of an IgG1 antibody, MEDI1912 (referred to here as IgG_WFL_)^35^. This recombinant human monoclonal antibody is specific for human nerve growth factor (NGF) and displayed significant aggregation and poor in vivo behaviour which was rectified in a variant containing three substitutions in the complementarity determining regions (CDRs) 1 (W35S and F36T, IMGT numbering^37,38^, Supplementary Fig. 1) and 2 (L64T) of V_H_, generating the variant referred to here as IgG_STT_^35^. The in vivo growth score of bacteria expressing each of these constructs was measured in a 48-well agar plate assay (Supplementary Fig. 2) over a range of ampicillin concentrations (0–140 µg mL^−1^) (Fig. 1c). For each protein, in vivo growth scores for the engineered variant with low aggregation (scFv_STT_, GCSF C3 and HEL4, Fig. 1d) (high in vivo growth score) is significantly enhanced relative to its aggregation-prone counterpart (scFv_WFL_, GCSF and Dp47d, Fig. 1d) (low in vivo growth score). These data validate the ability of the TPBLA to distinguish aggregation-prone proteins from their less aggregation-prone sequences over a range of different protein scaffolds.

**Fig. 2:**
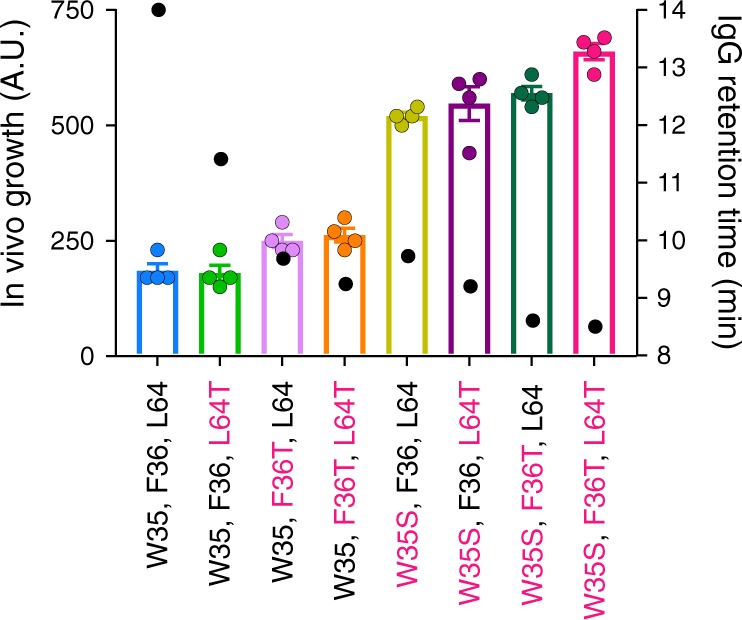
Comparison of the aggregation of WFL and its sequence variants in a scFv format in vivo and in an IgG1 format in vitro. Average in vivo growth score (bars, with individual experimental data shown as points) for scFv_WFL_ and scFv_STT_ together with their six combinatorial variants. Positions with the same amino acid as STT are highlighted in pink. Error bars represent s.e.m. (*n* = 4 biologically independent experiments). These data are overlaid with the HP-SEC retention times for the same variants reformatted as an IgG1 (black dots). Source data are provided as a Source Data file.

As the biopharmaceutical sector is currently dominated by IgGs, and many next generation therapies will also be based on this class of proteins or their derivatives, we focussed subsequent work on scFv_WFL_ and scFv_STT_. To assess the ability of the tripartite β-lactamase assay to differentiate between proteins with small changes in sequence, and to determine which of the amino acid substitutions (W35S, F36T or L64T) is responsible for the improved behaviour, the survival curves and in vivo growth scores for six variants that substituted W35S, F36T and L64T either individually or in combination were measured. The results showed that W35S largely endows aggregation resistance, followed by F36T, with L64T making little contribution (Fig. 2, Supplementary Fig. 3). Importantly, this insight can be achieved without the need to express and purify the proteins for biophysical analysis.

### In vivo scFv aggregation correlates with IgG1 aggregation

In order to use the TPBLA as a mAb developability screen it is essential that the minimal scFv constructs used in the assay yield similar aggregation propensities when reformatted as a full-length IgG. Consequently, each of the eight scFvs variants described above (WFL, WFT, WTL, WTT, SFL, SFT, STL and STT) were generated as IgG1 antibodies and their retention time on a high-performance size exclusion chromatography (HP-SEC) column was quantified (Fig. 2). HP-SEC is usually used in the biopharmaceutical industry to assess aggregation by quantification of monomer loss. As shown previously^35^, while IgG_STT_ has an elution time consistent with a monomeric IgG1 (∼8.5 min), IgG_WFL_ displays an asymmetric elution profile with a longer retention time (∼14 min) than expected based on monomer mass even in the presence of 125 mM arginine^39^. Consequently, the retention time was used in this study to assess the non-specific interactions/aggregation of this family of biologics. Overlaying the retention times for all eight variants with the in vivo assay scores shows an excellent correlation between an improvement in bacterial growth and a decrease in column retention time (Fig. 2). The interface between IgG_WFL_ dimers, formed en route to larger aggregates, has been shown previously (by chemical cross-linking and MS mapping) to be mediated by contacts between the V_H_ and V_L_ domains in different molecules^35^. Repeating these experiments for scFv_WFL_ and scFv_STT_, using increasing concentrations of the amine-specific cross-linker bis(sulfosuccinimydyl)suberate (BS^3^) showed that the majority of scFv_STT_ (99%) remained monomeric, whilst 45% of scFv_WFL_ was incorporated into higher-order oligomers (Supplementary Fig. 4a), consistent with both the TPBLA and HP-SEC data. Analysis of the dimers formed in scFv_WFL_ by mass spectrometry showed inter-protein cross-links between residues M0 (V_H_) and K66 (V_L_) and M0 (V_H_) and M0 (V_H_) (IMGT numbering), consistent with those formed for IgG_WFL_ (Supplementary Figs. 4 and 5), confirming conservation of the interactions formed in the initiating stages of aggregation. Together, these results show that the TPBLA allows the rapid assessment of the aggregation propensity of scFv fragments, which are maintained when inserted into an IgG1 scaffold.

### Evolving proteins with reduced aggregation propensity

**Fig. 3:**
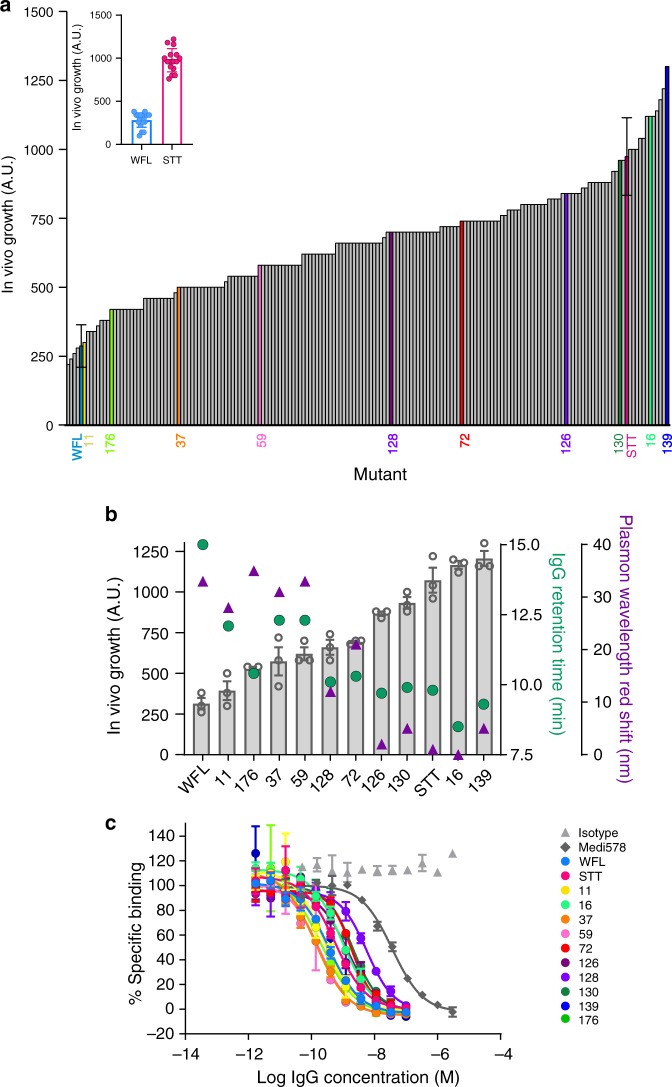
In vivo growth score of evolved βLa-scFv_WFL_ variants and the aggregation propensity and target affinity of ten selected variants in IgG1 format. **a** Ranked in vivo growth score of 185 variants (Inset shows error for controls βLa-scFv_WFL_ and βLa-scFv_STT_, data represent mean values ± s.d. (*n* = 15 biological repeats)). Ten variants across the rank (11, 176, 37, 59, 128, 72, 126, 130, 16 and 139) were selected and reformatted as full-length IgG1s for biophysical analysis. **b** HP-SEC retention time (green dots, longer times indicate greater interaction with column matrix) and AC-SINS (purple triangles, larger plasmon shifts correlate with greater self-association. *n* = 3 technical repeats. Note: error bars smaller than symbols (mean values)) of the ten selected variants in IgG1 format. These data correlate inversely with in vivo growth score (grey bars represent mean values, error bars represent s.e.m. *n* = 3 technical repeats). **c** Data used to calculate the IC_50_ values of binding of the ten evolved variants in IgG1 format to NGF determined using a homogeneous time-resolved fluorescence assay (HTRF). Data represent mean values ± s.d. (*n* = 3 technical repeats). Source data are provided as a Source Data file.

Having established that the TPBLA could be used in candidate selection, we next sought to use the assay as a screen for directed evolution in order to search for novel sequences able to ameliorate poor developability for candidates with promising therapeutic activity such as IgG_WFL_. To achieve this, genetic variation was introduced into the gene encoding scFv_WFL_ using error-prone PCR, before inserting the resulting library of sequences into the β-lactamase vector yielding a library of 1.3 × 10^6^ mutants (Methods). The DNA sequences of 57 variants in the naive library revealed an average mutational frequency of 8 amino acid substitutions per scFv. For screening, the plasmid DNA library of βLa-scFv_WFL_ variants (βLa-scFv_WFL_*) was transformed into *E. coli* SCS1 cells and plated onto agar containing 80 µg mL^−1^ ampicillin. At this antibiotic concentration, colonies should only grow if they express βLa-scFv_WFL_ variants that increase the expression of soluble and functional β-lactamase, compared with wild-type βLa-scFv_WFL_ (refer to Fig. 1 c). From the 315 colonies that could grow under this selection pressure, 185 variants were randomly selected and their in vivo growth score was measured, together with that of βLa-scFv_WFL_ and βLa-scFv_STT_. The resulting data (Fig. 3a) showed that 181 of these 185 variants displayed enhanced growth relative to WFL, with 12 having superior growth to the rationally engineered aggregation-resistant STT. To determine whether the in vivo growth score for these evolved variants also correlates with reduced aggregation propensity within an IgG1 scaffold, ten variants that spanned the rank order were converted to IgG1 molecules for further analyses. Molecules were selected sequentially across the rank (starting with 139, the best performing variant) by calculating in vivo growth scores separated by one standard deviation (s.d.) of the replicate error (βla-scFv_STT_ *n* = 16, s.d. = 130). For each of these values, the variant with the fewest substitutions relative to WFL was selected for further analyses in IgG format. This identified eight variants (11, 176, 59, 72, 126, 130, 16 and 139). Two further sequences (37 and 128) were selected for study as these were found to retain the original WFL residues (W35, F36 and L64) yet had improved in vivo growth score. The location and identity of each of the substitutions for these variants are shown in Supplementary Fig. 6. The aggregation propensity of these IgGs was then assessed using HP-SEC. As with the rationally engineered variants (Fig. 2a), a clear inverse correlation was observed between the retention time on HP-SEC and the in vivo growth score: antibodies with high in vivo growth scores exhibited shorter retention times reflecting reduced interactions with the column matrix (Fig. 3b). The aggregation properties of the ten evolved IgG variants, together with IgG_WFL_ and IgG_STT_, were also measured using AC-SINS (affinity-capture self-interaction nanoparticle spectroscopy)^40,41^. This method identifies self-association by an increase in the plasmon wavelength of gold nanoparticles upon their clustering induced by self-association of antibodies immobilised on their surface. Figure 3b shows an excellent direct correlation between the magnitude of the red shift in AC-SINS with the retention time by HP-SEC, adding further support to the ability of the TPBLA to select for sequences with reduced tendency to self-associate both as scFvs and as intact IgGs. Interestingly, as the majority of the evolved variants displayed similar thermal melting transitions to those observed for IgG_WFL_ (56 and 73 °C, assessed by differential scanning fluorimetry, DSF), no correlation was found between the aggregation propensity or the in vivo growth score with thermal stability (Supplementary Fig. 7, Supplementary Table 1).

The application of a single selection pressure may result in increased aggregation-resistance at the expense of target affinity, akin to affinity/stability trade-offs^29^. To assess this possibility, IC_50_ values for the cognate antigen NGF^35^ were measured for each of the ten evolved IgG variants by a competition binding assay monitored by fluorescence, and the results compared with IgG_WFL_, IgG_STT_ and MEDI578 (the parent antibody prior to affinity maturation into MEDI1912^35^ (Fig. 3c, Supplementary Table 2)). The results showed that all of the evolved antibodies maintain higher affinity to NGF than MEDI578, demonstrating that all variants retain functional activity, with no correlation between IC_50_ and in vivo growth score values (Supplementary Fig. 8)^35^.

### Mutation hotspots identify localised frustration within IgGs

**Fig. 4:**
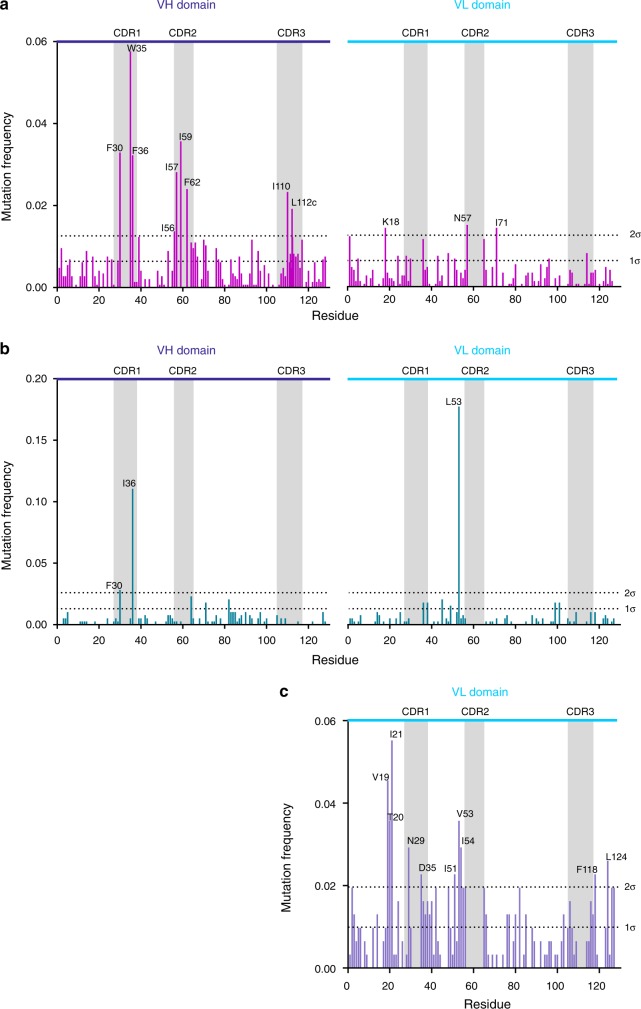
Mutation frequency profile of evolved antibody fragments. CDRs are highlighted as grey rectangles and V_H_ and V_L_ domains are labelled. **a** Mutational frequency of the screened scFv_WFL_* library reveals 12 residues with a mutational frequency greater than two standard deviations from the average value (2σ). Nine occur in V_H_ and three in V_L_. **b** The mutation frequency profile for screened scFv_Li33_ reveals only three sites with a mutational frequency >2σ. **c** The mutational frequency of the evolved V_L_ domain, JTO, reveals 10 residues with a mutational frequency >2σ. All profiles use IMGT numbering. The cumulative mutational frequency is normalised to 1 for each dataset. Residues showing high mutational frequencies (>2σ) are labelled in each case. Datasets are pooled from two independent experiments. Source data are provided as a Source Data file.

Analysis of the mutational frequency of individual residues within the aggregation-resistant scFv sequences enabled a protein-specific profile of residues that might contribute to aggregation to be generated. Such ‘hotspot’ residues represent ideal targets for mutation to improve bioprocessability of the sequence when reformatted as an IgG, or to improve soluble expression of proteins more generally. The mutational-frequency profile across the V_H_ and V_L_ domains of the library (βLa-scFv_WFL_*) was constructed from the sequences of all 315 variants that grew under the selection pressure of 80 µg mL^−1^ ampicillin (Fig. 4a). This analysis revealed 12 hotspot residues with a mutational frequency significantly higher (>2 s.d.) than the mean (labelled by residue in Fig. 4a). Nine of these residues, which are all hydrophobic or aromatic, lie in the V_H_ domain, and are clustered in the CDR regions: F30, W35, F36 (CDR1), I56, I57, I59 and F62 (CDR2), and I110 and L112c (IMGT numbering) (CDR3). The remaining three hotspot residues lie in the V_L_ domain (K18, N57 and I71). The chemical identity of the most frequently selected residue, and whether a particular amino acid residue is enriched relative to all other residues possible via a single-base-pair change, was also assessed (Table 1). Interestingly, the hotspot residues in V_H_ (most of which are solvent exposed and hydrophobic) tended to be substituted with more hydrophilic residues (Ser and Thr), while the hotspot residues in the V_L_ domain which were initially charged (K18), hydrophilic (N57) or hydrophobic (I71) were substituted with polar or other charged amino acids.

**Table 1:** Summary of the 12 most frequently substituted residues after directed evolution of the scFv_WFL*_ library.

Residue	RSA^a^	Most frequently observed aa substitution	Mutation frequency of most often observed mutation	Expected frequency of most often observed mutation^b^	Amino acid substitutions observed^c,d^	Available residues with single DNA base change^d^
F30	0.07	S	0.81	0.41	SL**P**V	IVLFCSY
W35	0.63	R	0.93	0.90	RG	LCGSR
F36	0.66	S	0.60	0.41	SL(V**P**)(I**T**)	IVLFCSY
I56	0.09	V	0.50	0.43	VT(LF)	IVLFMTSN
I57	0.01	T	0.66	0.44	TNV**A**	IVLFMTSN
I59	0.44	T	0.60	0.43	TNVF	IVLFMTSN
F62	0.45	S	0.60	0.43	SLY	IVLFCSY
I110	0.19	T	0.82	0.43	TV(LFM)	IVLFMTSN
L112c	0.65	P	1.00	0.83	P	IVLFPHR
K18_LC_	0.81	E	0.52	0.44	ERNQ	ITEQNKR
N57_LC_	0.18	D	0.73	0.43	DS**G**	ITSYHDNK
I71_LC_	0.24	T	0.62	0.43	TVN	IVLFMTSN

^a^RSA = relative surface area (0 = completely buried residue, 1 = maximally solvent exposed residue (see Methods)).

^b^Expected frequency calculated using the mutational bias found in the naive library.

^c^Residues are listed in decreasing mutational frequency with brackets indicating residues with equal frequency of mutation. Substitutions shown in bold are due to two base-pair changes in the DNA codon.

^d^Amino acids are listed in decreasing hydrophobicity (left to right) using the Kyte-Doolittle scale^57^.

In order to understand whether the mutation frequency profile for scFv_WFL_ was specific for this scFv sequence, or simply reflected innate frustration of the Ig-fold itself, we performed the same directed evolution screen on two other IgG scaffolds: a second industrially-derived scaffold (a scFv variant of the anti-LINGO-1 mAb, Li33^42^) and a λV6-57 V_L_ domain (JTO) isolated from a patient with multiple myeloma with tubular cast nephropathy^43^ (Supplementary Figs. 9a and b). The resultant mutational-frequency profiles (Fig. 4b and c), from 140 and 75 DNA sequences, respectively, contrast markedly and also are distinct to that for scFv_WFL_ (Fig. 4a). For JTO, the TPBLA reveals sequence-wide frustration, with clusters of frequently mutated residues (>2σ) observed both within, or directly flanking, its CDRs, as well as in the framework region (notably involving residues 19–21 and 51–54 and the C-terminal region). In accord with recent work by Rennella et al.^44^, this apparently non-specific profile may reflect the fact that the aggregation of this domain is driven from the unfolded state by interactions between APRs throughout the structure. In this case, the TBPLA may select for sequences with both decreased aggregation propensity and increased local or global thermodynamic stability (which decreases the population/lifetime of solvent exposed APRs). In light of this, the most frequently mutated hotspot was found in β-strand B (residues 19–21, Fig. 4c), highlighting this region as a particularly important driver of aggregation as reported previously^44^.

By contrast, both to this sequence-wide effect and the relatively localised profile for scFv_WFL_, scFv_Li33_ showed minimal frustration. Only three residues, F30 and I36 in V_H_ (most commonly substituted with S and T, respectively) and L53 in V_L_ (most commonly substituted with P), exhibited substitution frequencies significantly greater (>2 s.d.) higher than the mean. The difference in the profiles of scFv_WFL_ and scFv_Li33_ is remarkable, given the similarity of their framework regions (66.5% similarity and 48.2% identity (Supplementary Fig. 10)), but may be expected as their poor developability has been ascribed to different mechanisms: aberrant CDR-CDR (WFL)^35^ and CDR-constant region (Li33)^42^ interactions. As the TPBLA employs scFvs, it cannot detect aberrant CDR- constant region interactions, and given that Li33’s solubility depends critically on the type of IgG scaffold^42^, we hypothesised that the dominant evolutionary pressure in the TPBLA for this sequence may be thermodynamic stability, rather than its innate aggregation propensity, as was also previously observed for the soluble globular protein Im7 using this assay^34^. To test this hypothesis, the scFv sequences for Li33, Li33_I36T_, Li33_Y88D_ and Li33_L53P_ (the single point variants with the highest in vivo growth score, Supplementary Fig. 9) were grafted into an IgG1 scaffold. Surprisingly, no significant changes in thermal stability were detected between the wild-type and evolved Li33_I36T_, Li33_Y88D_ variants, whilst a single broad transition was observed for Li33_L53P_ (Supplementary Fig. 11, Supplementary Table 1). Instead, small but significant reductions in self-association monitored by AC-SINS (Supplementary Fig. 12a) over wild-type Li33 were observed for I36T and L53P. In addition, L53P and Y88D showed increased solubility relative to wild-type as assessed by a polyethylene glycol (PEG) precipitation assay (Supplementary Fig. 12b). These data suggest that the TPBLA is able to identify (and resolve) specific problematic residues between proteins with identical topologies and highly similar sequences and does not simply identify scFvs with increased thermal stability. Together, these results demonstrate the power of the TPBLA to develop new understanding of the molecular determinants of aggregation associated with proteins of relevance for bioprocessing, as well as those associated with protein misfolding diseases.

### Comparison of mutational hotspots with in silico predictions

**Fig. 5:**
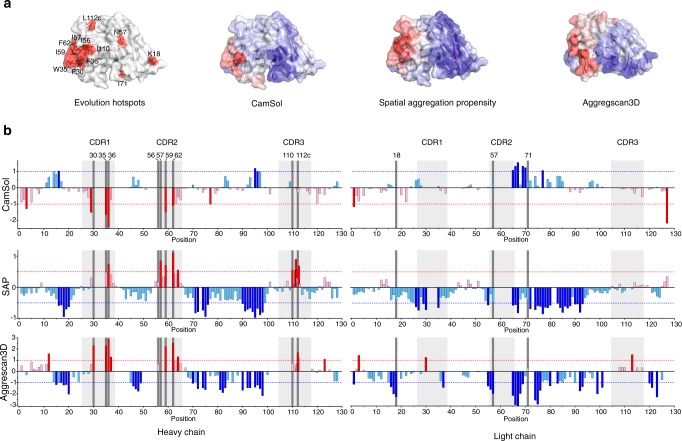
Comparison of computational predictors of aggregation with the evolved mutational hotspots for WFL. **a** Comparison of evolution hotspots for scFv_WFL_, with predictions based on (left to right) structurally corrected CamSol^18^, SAP^19^ or Aggrescan3D^45^. Insoluble/aggregation-prone and soluble/non-aggregation-prone regions are shown on a surface model of the protein (created from PDB 5JZ7^35^) in red and blue, respectively. **b** Computational prediction of insoluble and/or aggregation-prone sequences of scFv_WFL_ for (top to bottom) structurally corrected CamSol, where +1 indicates soluble and −1 indicates insoluble (dotted lines); SAP (using a 10 Å radius), where values >0.5 and <−0.5 are significant; and Aggrescan3D, where values >1 and <−1 are significant. In each plot the significance values are highlighted by dotted lines and colours are as in (**a**). Dark grey vertical bars denote evolution hotspot residues and light grey boxes highlight CDRs. Residues are numbered according to IMGT numbering. Supplementary Fig. 13 shows an expanded view of residues 111–112. Source data are provided as a Source Data file.

Several in silico approaches have been developed to identify residues/sequences with poor solubility (e.g. structurally corrected Camsol^18^), or high aggregation propensity (e.g. Aggregscan3D^45^ and SAP^19^). Comparison of the location of the sequence hotspots identified here for scFv_WFL_ by directed evolution, with those predicted based on these algorithms are shown in Fig. 5a, b, Supplementary Fig. 13 and Supplementary Table 3. The results portray the complexity in determining protein aggregation based on predictions alone. Each algorithm detected at least one of the insoluble or aggregation-prone residues in CDR1 and CDR2 of scFv_WFL_ that form the large hydrophobic patch shown previously^35^, and recapitulated here for scFv_WFL_, to be involved in the aggregation interface. The identity of the residues involved, however, varied between algorithms. SAP and Aggrescan3D also identified a third hotspot-cluster in CDR3. In addition, each in silico method highlighted additional residues in V_H_ that were not identified by directed evolution, and no in silico method identified any of the hotspot residues in V_L_. In total the three algorithms highlighted 26 residues as potential positions in which aggregation could be suppressed by mutation, including eight of the 12 most frequently mutated residues identified here by directed evolution. However, only three residues are flagged by all three algorithms (Supplementary Fig. 14).

Since the variants of scFv_WFL_ generated by evolution each contain several mutations (for example see Supplementary Fig. 6b), the importance of individual amino acid substitutions to the properties of the proteins remained unknown. To examine the relative importance of each individual substitution and to determine how these values relate to in silico predictions, the in vivo growth score for βLa-scFv_WFL_* variants containing the most common amino acid substitution in each of the 12 hotspot residues was measured (Supplementary Fig. 15). While no single substitution was found to match the in vivo growth score for STT (690 ± 8 A.U.), F62S, a residue not mutated in rational design of STT^35^, achieved 91% of this enhancement (643 ± 27 A.U.). By contrast, F36, which was mutated to Thr in the rational design of IgG_STT_, only achieved 15% of the enhancement when mutated to Ser in our assay (scores for WFL and F36S were 172 ± 53 A.U. and 250 ± 9 A.U., respectively). Quantifying the effect of single substitutions also did not improve the correlation with in silico methods. For example, only three residues (F36, I59 and F62) are flagged by all three computational methods (Supplementary Fig. 14), yet these vary considerably in their in vivo growth score (ranked 10th, 5th and 1st, respectively (Supplementary Fig. 15)). Overall, while there is some agreement between in silico approaches and the TPBLA, the identity of the problematic residues determined experimentally and predicted computationally varies considerably (Supplementary Table 3). Hence, identification of the key residues to target by rational engineering would be difficult using a multi-algorithm approach, highlighting the power of using evolution to find solutions to the problem of aggregation.

## Discussion

Split β-lactamase assays have been used previously as a proxy for several characteristics including protein–protein interactions^46^, a marker for gene expression^47^, and for selecting open reading frames^48^. These assays exploit β-lactamase’s stability, the presence of permissive grafting sites in its structure, and the potential for high throughput screening via colorimetric assays. Here, we have shown the ability of the versatile tripartite β-lactamase platform^32,34,49^ to distinguish aggregation-prone variants of diverse biopharmaceutically-relevant protein scaffolds from their more aggregation-resilient counterparts. In contrast with other in vivo systems for studying protein aggregation^50,51^, this assay has the advantage that fusion proteins are expressed in the periplasm of *E. coli*, allowing the formation of disulfide bonds, such as those found in IgGs and their derivatives. Most importantly, no perturbant such as increased temperature, pH or chemical denaturant is used to accelerate aggregation, allowing identification of sequence characteristics that trigger innate (unaccelerated) aggregation pathways. Furthermore, this assay has broad utility as it is agnostic to the underlying mechanism of aggregation (e.g. unstructured peptides with a high propensity for amyloid formation as well as for globular proteins that self-assemble through a variety of mechanisms). We have shown that the aggregation propensity driven by the self-association of IgG_WFL_ is largely determined by the Fv region, as IgG1 and scFv homologues yield the same rank order of aggregation propensity judged both within the βLa fusion, and as purified IgGs (Fig. 2a). As scFvs are commonly reformatted into IgG scaffolds, and scFv formats are frequently used in phage or other display systems, the assay could readily be integrated into the development pipeline to identify developable sequences directly after discovery and affinity maturation. The assay is amenable to any protein displayed as a single chain, and hence could be used to optimise a wide variety of biologics, including dAbs, scFabs, scFc (with co-expression of these allowing the detection of Fab:Fc interactions) and bispecifics (in scFv format) all of which are poorly characterised in terms of developability relative to platform IgGs.

We have also shown here the power of the TPBLA combined with directed evolution to rectify problematic sequences, and to identify mutational hotspots that limit protein behaviour (due to a variety of mechanisms) in both the variable and framework regions. Here, we took a previously characterised IgG with known development issues^35^, IgG_WFL_, and engineered new sequences (Fig. 3a) with reduced self-association as measured by HP-SEC and AC-SINS (Fig. 3b, c). It is notable that the sequence of the rationally engineered IgG_STT_ was not isolated during screening. The best performing evolved variant involved substitution of only one of these residues (F36), demonstrating the advantages of directed evolution and selection over rational approaches. Screening a randomised scFv library of a second industrially-derived sequence (Li33) identified substitutions in different hotspots. These substitutions were found to improve solubility in the context of an IgG1 scaffold (Supplementary Fig. 12), demonstrating its broad utility. In this regard, it is intriguing that the mutational-frequency profile for the ‘synthetically-derived’ sequences of WFL and Li33 differ significantly from one optimised for humoral immunity, examined here using JTO. A complex mutational profile is observed for the latter, which may reflect both the selective pressure to increase thermodynamic stability and minimise APRs (whose identity found here using the TPBLA correlates with experimental results and in silico predictions^44^). By contrast, the majority of hotspot residues identified for both scFv_WFL_ and scFv_Li33_ are located in, or close to, the CDRs. Given the importance of the CDRs in determining epitope binding affinity, this is unsurprising and, at first glance, may appear to be problematic for the maintenance of a successful candidate profile. We have shown here, however, that binding affinity can be maintained concomitantly with a significant improvement in aggregation performance, at least for variants of IgG_WFL_, presumably because only a subset of CDR residues are directly involved in epitope binding^35^. Indeed, the crystal structure of the scFv of the IgG_MEDI578_ (the parental sequence of IgG_WFL_) in complex with its ligand^35^ shows direct interaction of only 6 of the 16 residues in CDR1 and CDR2, with CDR3 making 13 out of a total of 22 contacts to NGF.

Despite the ability of the TPBLA to generate candidate sequences with greatly improved properties, it remains challenging to determine how the different amino acid substitutions introduced actually ameliorate aggregation. This arises because aggregation results from a complex interplay of properties that includes kinetic and thermodynamic stability, the number and position (solvent accessibility) of aggregation-prone regions, and local dynamics that may expose those regions. Analysis of the substitutions made here for scFv_WFL_ in the context of their location in the protein (Table 1) allows the cause of the liability to be putatively assigned. For example, 10 of the 12 hotspot residues found for scFv_WFL_ are hydrophobic/aromatic in nature and all were substituted with more hydrophilic residues, consistent with the mechanism of aggregation suggested previously for this protein^35^. In accordance with this hypothesis, three different algorithms that predict solubility and aggregation propensity of amino acid sequences within structured^18^ and dynamic protein domains^19,45^, identify the same region. These algorithms, however, yield different predictions, confusing the choice of residues to mutate in any rational approach to improve protein behaviour.

The ability of the TPBLA to quantify aggregation propensity in the absence of protein purification engenders its use as a fundamental research tool. Firstly, it allows optimisation of protein expression for experimentally intractable proteins (e.g. aggregation-prone or insoluble proteins). Secondly, using the TPBLA as a screen for deep mutational scanning^52^ would allow areas of sequence frustration (i.e. destabilisation due to functional constraints or aggregation propensity) to be mapped and their underlying mechanisms of aggregation to be better understood, enabling their modification using rational engineering approaches. A fundamental understanding of biopharmaceutical aggregation during manufacture and storage is still lacking more than 30 years since the first introduction of IgGs into the clinic. We have shown here that the TPBLA is a powerful method by which to identify (using the TPBLA alone), or re-engineer (using the TPBLA as a directed evolution screen) inherently manufacturable proteins. Combining the TBPLA with the approaches described above may thus be of enormous practical benefit to create proteins with improved behaviour, and when combined with evolution methods, may be able to provide the mechanistic understanding needed to apply a true quality-by-design approach to biopharmaceutical discovery and development.

## Methods

### Construction of β-lactamase fusions

Synthetic DNA sequences (purchased from Eurofins Genomics) encoding GCSF, scFv_WFL_ and scFv_Li33_, and their variants, were inserted in-frame into the 28-residue G/S linker (previously inserted between residues 196 and 197 of TEM-1 β-lactamase^34^) in the pMB1-βla-linker plasmid via a 5′ (XhoI) and 3′ (BamHI) restriction site. Ligation products were transformed into *E. coli* SCS1 cells (Stratagene) and the cells grown on agar plates containing 10 µg mL^−1^ tetracycline. The identity of the resulting clones was verified by DNA sequencing (DNA and amino-acid sequence of the βla-linker and the test protein variants used are listed in Supplementary Tables 4 and 5).

### In vivo growth assay

MCD_GROWTH_ (maximal cell dilution allowing growth) assays were performed in sterile 48-well LB agar plates (Greiner Bio-One, cat. 677102) prepared prior to the assay. Tetracycline (10 µg mL^−1^ final concentration) and filter-sterilised L-arabinose (final concentration of 0.075% (w/v) for scFv_WFL_/scFv_Li33_ or 0.1% (w/v) for JTO) were added to 100 mL of sterile 1.5 % (w/v) LB agar cooled to <50 °C. Three hundred microlitres of this solution was added into each of the first 6 wells (first row) of the 48-well plates. Ampicillin (10 mg mL^−1^ stock) was then added to the LB agar stock to give the required concentration for the next row of wells. This procedure was repeated until the plate contained 8 rows of LB agar containing increasing concentrations of ampicillin. β-lactamase-test protein constructs were screened over an ampicillin range of either 0–140 μg mL^−1^ (20 μg mL^−1^ increments) or 0–280 µg mL^−1^ (40 µg mL^−1^ increments). Agar plates were left to set in a sterile environment.

A single colony of fresh *E. coli* SCS1 cells (Stratagene) transformed with the appropriate plasmid was used to inoculate 5 mL sterile LB containing 10 µg mL^−1^ tetracycline. Cultures were incubated overnight at 37 °C with shaking (200 rpm). One millilitre of overnight culture was used to inoculate 100 mL sterile LB containing 10 µg mL^−1^ tetracycline and grown at 37 °C (shaking at 200 rpm) until an OD_600_ of 0.6 was reached. Expression of the β-lactamase fusion construct was induced by the addition of filter-sterilised arabinose at a final concentration of 0.075% (w/v) (scFvs) or 0.1% (w/v) (JTO). Cultures were incubated for a further 1 h then serially diluted 10-fold into sterile 170 mM NaCl solution. Three microlitres of each dilution was then spotted onto the pre-prepared 48-well agar plates. The plates were incubated at 37 °C for 18 h and the MCD_GROWTH_ was determined for each ampicillin concentration by visual inspection.

A single value from each MCD_GROWTH_ assay, illustrative of the effect of each scFv on bacterial growth, was calculated from the area under the MCD_GROWTH_ curves as a sum of the areas of 7 trapezia using Eq. (1), where *A*_curve_ is the total area under the curve, and *x*_*i*_ and *y*_*i*_ are the *x*-axis and *y*-axis values at each concentration of ampicillin.

1 $$A_{{\mathrm{curve}}} = \sum_{i = 1}^7 {\frac{{y_i + y_{i + 1}}}{2}} \times \left( {x_{i + 1} - {\mathrm{x}}_i} \right)$$

### Construction and expression of IgGs

scFv variants chosen for further study were reformatted into the TM-YTE IgG1 backbone by cloning eukaryote codon-optimised V_H_ and V_L_ domains into human TM-YTE IgG1 heavy chain and light chain expression vectors^53^. The plasmids were co-transfected into HEK293/EBNA mammalian cells (Invitrogen, Catalog no. R620-07) for expression and IgG proteins purified from the culture medium using Protein A chromatography.

### High-performance size exclusion chromatography (HP-SEC)

HP-SEC was performed using an Agilent 1100 series HPLC fitted with a TSK SWXL HPLC guard column (Tosoh Bioscience) and TSK-GEL G3000SW_XL_ HPLC column (Tosoh Bioscience). Fifty microlitres of IgG at 1 mg mL^−1^ in Dulbecco’s Phosphate Buffered Saline (D-PBS) (Sigma-Aldrich) was injected at a flow rate of 1 mL min^−1^ using 0.1 M sodium phosphate, 0.1 M sodium sulphate, pH 6.8 as the mobile-phase buffer.

### Chemical cross-linking

scFvs (15 µM) were dialysed into 100 mM sodium phosphate buffer, pH 7.4 and cross-linked with a mixture (50:50) of d_0_-BS3 and d_4_-BS3 (Thermo Scientific, UK) at different molar excesses: 50×, 100×, 200× and 500×. The cross-linking reaction was left for 30 min at 25 °C before being quenched with Tris.HCl, pH 8 (50 mM final concentration). The cross-linked and non-cross-linked samples were then resolved using SDS-PAGE and the cross-linked dimer bands, along with cross-linked and non-cross-linked monomer, were excised from the gel for in-gel trypsin digestion. The gel pieces were subjected to three repeat rounds of hydration and dehydration with 25 mM ammonium bicarbonate, pH 7.8 and 50% (v/v) acetonitrile/25 mM ammonium bicarbonate, respectively. Samples were then treated with 10 mM DTT and the cysteine residues subsequently alkylated with 55 mM iodoacetamide. The gel pieces were dehydrated again using 50% (v/v) acetonitrile/25 mM ammonium bicarbonate before being re-hydrated with a 0.1 µg/µL trypsin solution and incubated for 18 h at 37 °C. Digested peptides were recovered from the gel by subjecting the gel pieces to four repeat rounds of dehydration with 60% (v/v) acetonitrile/5% (v/v) formic acid. Extracted peptides were then concentrated before LC-MS/MS analysis. Densitometry calculations were performed using ImageJ.

### LC-MS/MS analysis of extracted cross-linked peptides

Extracted peptides were analysed on a nanoAcquity LC system connected on-line to a Synapt G2-Si mass spectrometer (Waters Ltd., Wilmslow, UK). One microlitre of extracted peptide samples were injected onto an Acquity M-Class column (C18, 75 µm × 150 mm) (Waters Ltd., Wilmslow, Manchester, UK) and subsequently separated by a 1–50% gradient elution of solvent B (0.1% (v/v) formic acid: acetonitrile) in solvent A (0.1% (v/v) formic acid in water) over 60 min at a flow rate of 0.3 µL min^−1^. The instrument was operated in positive ion mode using collision-induced dissociation (CID) for fragmentation of selected ions. Data dependant MS/MS experiments were conducted in the trap region of the instrument using a 1-s scan with the five most intense ions being selected for fragmentation over a 350–2000 *m*/*z* window. Fragmentation of less abundant cross-linked ions was achieved through manual inclusion in sequential acquisitions after analysis of the MS and MS/MS data. The data were analysed using the MassLynx software (version 4.1) and StavroX (version 3.6.0.1).

### Cross-linking data analysis

Data were imported into PEAKS studio (version 10) and exported as MGF files, to then be imported and analysed by StavroX. Due to the significantly lower levels of dimer formed for scFv_STT_, the 200x cross-linked sample was used while the 50x cross-linked sample was used for scFv_WFL._ The data were searched against the protein sequences for scFv_WFL_ and scFv_STT_. StavroX parameters used for searching were as follows: K and R protein cleavage sites (with 2 and 1 potential missed cleavages, respectively), fixed modification of C to B (cysteine to carboxyamidomethylcysteine) and variable modifications of M to m (methionine to oxidised methionine) with a maximum of two variable modifications per peptide. The non-deuterated and deuterated BS3 cross-linker was added to StavroX, C_8_H_10_O_2_ (138.07 Da) and C_8_H_6_D_4_O_2_ (142.09 Da), respectively. To include all potential cross-links, the site specificity was set as lysine (K) for peptide 1 and to lysine (K), serine (S), threonine (T) or tyrosine (Y) residues, as well as including the N-terminal amine 54 for peptide 2. Mass tolerances were set as 3.0 ppm for the precursor ions and 0.8 Da for fragment ions with mass limits of 200–8000 Da. Low-precise scoring was used with an false detection rate cut-off of 5% and a score cut-off of 10. A decoy database was generated by shuffling the sequence while keeping the protease sites.

Manual data validation was achieved using a comparative approach searching for unique peptides from the digested dimer bands. Cross-linked peptides were readily identified from the doublet peak (Δ4 Da) formed by use of a deuterated and non-deuterated cross-linker.

### DNA library synthesis

The Diversify PCR Random Mutagenesis Kit (Takara) was used to synthesise a scFv megaprimer (error rate of 8.1 (WFL), 2.7 (Li33) or 5.8 (JTO) mutations per 1000 bp), using forward (5ʹ-GTGGTGGTGGCTCGA) and reverse (5ʹ-AACCGCTCCCGGATC) primers that anneal to the Gly/Ser linker regions up- and down-stream of the scFv sequence. The product was purified on a 1% (w/v) agarose gel and the desired band was excised and purified using Qiagen Gel Extraction Kit, according to the manufacturer’s instructions. To prevent expression of wild-type βLa-scFv_WFL_ after ligation, a ‘stop template’ plasmid was created. To this end, two stop codons were inserted into β-lactamase (amino acid positions 109 & 110, Supplementary Table 6) in the pMB1-βLa-scFv_WFL_ plasmid using the Q5 Site-Directed Mutagenesis Kit (NEB). A ten-fold excess of scFv megaprimer was added to the βLa-scFv_WFL_ stop template and splicing performed using the QuikChange Lightning Site-Directed Mutagenesis Kit (Agilent). Two microlitres DpnI was then added to each reaction (1 h, 37 °C) to remove template DNA. The product was purified using Qiagen PCR Purification Kit and 2 µL was used to transform TG1 Electrocompetent cells (Lucigen) by electroporation (2.5 kV field strength, 335 Ω resistance and 15 µF capacitance). Following recovery, cells were plated onto pre-prepared LB bioassay agar plates containing 10 μg mL^−1^ tetracycline and incubated overnight at 37 °C.

Single colonies were picked for sequence analysis before the remaining colonies were removed from the bioassay plates by addition of 10 mL LB medium and scraping off. The culture was centrifuged (10 min, 5000 × *g*) before DNA extraction using the Qiagen Midiprep Kit, according to the manufacturer’s instructions.

### Evolution assay

Directed evolution bioassay assay plates were prepared containing 2.5% (w/v) LB, 1.5% (w/v) agar, 10 µg mL^−1^ tetracycline, 0.075% (scFvs) or 0.1% (JTO) (w/v) arabinose and either 80 µg mL^−1^ (WFL) or 140 µg mL^−1^ (Li33 and JTO) ampicillin. SCS1 Supercompetent Cells (Agilent) were thawed on ice for 10 min and 50 µL cells transferred to a 14 mL round-bottomed transformation tube. Two microlitres of the prepared library plasmid DNA (100 ng µL^−1^) was added to the cells and incubated on ice for 30 min before heat shocking at 42 °C for 45 s. After 5 min incubation on ice, 950 µL SOC medium was added to cells and incubated (37 °C, 200 rpm) for 1 h. Three millilitres SOC medium was then added to the cells along with 10 µg mL^−1^ tetracycline. Cells were incubated for 4 h and β-lactamase expression then induced with 0.075 % (w/v) arabinose. Cells were then incubated (37 °C, 200 rpm) for 1 h. The culture was spread onto the prepared assay plates and incubated overnight at 37 °C.

### Affinity-capture self-interaction nanoparticle spectroscopy (AC-SINS)

AffiniPure goat anti-human IgG Fcγ Fragment specific (IgGα-Fc) and ChromePure Goat IgG, whole molecule (IgG_WHOLE_) (Jackson ImmunoResearch) were buffer exchanged into 20 mM potassium acetate, pH 4.3 and diluted to 0.4 mg mL^−1^. Nine millilitres of citrate-stabilised 20 nm gold nanoparticles (Expedeon) were incubated with 600 µL IgGα-Fc and 400 µL IgG_WHOLE_ for 2 h at room temperature. Nanoparticles were blocked with 0.1 µM 2000 MW thiolated PEG (Sigma-Aldrich) at room temperature for 1–2 h. Nanoparticles were concentrated to 800 µL in siliconised Eppendorf tubes (VWR) and stored at 4 °C. Forty-five microlitres of 50 µg mL^−1^ antibody samples were mixed with 5 µL nanoparticle solution and incubated at room temperature for 30 min. The mixture was transferred to a 384-well polystyrene UV transparent plate (Thermo Scientific), and the absorbance read from 400 to 700 nm in 1-nm increments. The maximum absorbance was determined (the plasmon wavelength) and the redshift in plasmon wavelength compared with nanoparticles in the absence of antibodies was then calculated by subtracting one from the other.

### Epitope competition assay

The relative affinity of the IgG_WFL_ variants for NGF was established using a homogeneous time-resolved fluorescence (HTRF) epitope competition assay. The assay determines relative affinity by measuring the reduction in binding of biotinylated NGF (R&D Systems (256-GF, biotinylated in-house)) to DyLight650-labelled IgG_WFL_ in the presence of increasing concentrations of test IgG. Binding of DyLight650-labelled IgG_WFL_ to biotinylated NGF is detected by FRET between streptavidin Europium cryptate (CisBio), which binds biotinylated NGF and the DyLight650 conjugated to the IgG. Fluorescence was measured on a PerkinElmer EnVision plate reader with the following settings: 100 flashes, delay 70, cycle 2000, Excitation UV2 (TRF) 320 nm, Emission APC 665 (Bandwidth 7.5 nm), Emission Rhodamine 590 (Bandwidth 20 nm), mirror D400/630. The HTRF ratio is calculated by Eq. (2) and the %DELTA F is calculated by Eq. (3):

2 $$\frac{{665\,{\mathrm{nm}}\,{\mathrm{emission}}}}{{590\,{\mathrm{nm}}\,{\mathrm{emission}}}} \times 10,000$$

3 $$\frac{{({\mathrm{Sample}}\,{\mathrm{ratio}}-{\mathrm{negative}}\,{\mathrm{control}}\,{\mathrm{ratio}})}}{{{\mathrm{Negative}}\,{\mathrm{control}}\,{\mathrm{ratio}}}} \times 100$$

### Differential scanning fluorimetry

Twenty microlitres of 0.52 mg mL^−1^ antibody solution in PBS was added to a white PCR plate (BioRad). SYPRO Orange protein gel stain (5000× stock, Invitrogen) was diluted to 40× in distilled H_2_O prior to addition of 5 µL to each well. The plate was sealed, and melt curves obtained on a BioRad CFX96 Real-Time PCR system (20–95 °C, increments of 0.2 °C per min and hold time of 10 s) by measuring fluorescence intensities using the FRET channel with excitation from 450 to 490 nm and detection from 560 to 580 nm.

### Relative surface accessibility (RSA)

RSA values were calculated by taking the absolute solvent accessible surface area for the residue in the model of the structure of scFv_WFL_ (created by mutating PDB 5J7Z^35^ using Pymol 2.1.0) and dividing it by the maximum possible area for the amino acid type as described by Miller et al.^54^.

### Poly(ethylene glycol) (PEG) precipitation assay

A 40% (w/v) PEG 10,000 (Sigma) solution was prepared in PBS and corrected to a pH of 7.0. PEG solution, PBS and 20 µL of IgG stock solution were combined to achieve a PEG concentration range of 0–10% (w/v) and final IgG concentration of 0.5 mg mL^−1^ in a 96-well plate in triplicate. Plates were sealed with adhesive sealing film and incubated at 4 °C for 24 h. After incubation, samples were thoroughly mixed in their respective wells before 2 µL of each sample was transferred to a Lunatic plate for turbidity measurement at 500 nm on a Lunatic (Unchained Labs). Turbidity of buffer only controls was subtracted from final readings.

### In silico aggregation predictors

A model of the structure of scFv_WFL_ (described above) was used. The webserver for CamSol^18^ was used to generate a structurally corrected profile at pH 7 with a 10 Å patch radius to identify soluble and insoluble amino acids located at http://www-vendruscolo.ch.cam.ac.uk/camsolmethod.html. Aggrescan3D 2.0^45^ server was used to predict aggregation propensity located at http://biocomp.chem.uw.edu.pl/A3D2/. Predictions were made in dynamic mode with a 10 Å radius, and stability calculation option was selected, using FoldX^55^ to optimise input structure. Spatial aggregation propensity (SAP) calculations were performed using CHARMM^56^ simulations and method described by Chennamsetty et al.^19^ using a 10 Å radius.

### Reporting summary

Further information on research design is available in the Nature Research Reporting Summary linked to this article.

## Supplementary information

Supplementary Information

Reporting Summary

## Data Availability

The datasets generated during the current study are available in the University of Leeds data repository (10.5518/739) and the Source Data file. The source data underlying Figs. 1c, 2, 3, 4 and 5 and Supplementary Figs. 3, 7, 9, 11, 12 and 15 are provided as a Source Data file. All other relevant data are available from the authors upon reasonable request.

## Source data

Source Data
