# Supplementary material for: An in vivo platform to select and evolve aggregation-resistant proteins
Source: Nat Commun. 2020 Apr 14;11:1816. doi: 10.1038/s41467-020-15667-1 (PMC7156504; doi:10.1038/s41467-020-15667-1)
Supplement: Supplementary file 1 — Supplementary Information [file 41467_2020_15667_MOESM1_ESM.pdf]

An *in vivo* platform to select and evolve aggregation resistant proteins

Supplementary Information

Jessica S. Ebo[1, 2, †], Janet C. Saunders[1,3,†], Paul W. A. Devine[1,3], Alice M. Gordon[1,2], Amy S. Warwick[1,2], Bob Schiffrin[1,2], Stacey E. Chin[3], Elizabeth England[3], James D. Button[3], Christopher Lloyd[3], Nicholas J. Bond[3], Alison E. Ashcroft[1,2], Sheena E. Radford[1,2], David C. Lowe[3]*, David J. Brockwell[1,2]*

† These authors contributed equally to this work

*Corresponding authors david.lowe@astrazeneca.com and D.J.Brockwell@leeds.ac.uk

[1]Astbury Centre for Structural Molecular Biology, University of Leeds, Leeds, LS2 9JT, UK

[2]School of Molecular and Cellular Biology, Faculty of Biological Sciences, University of Leeds, Leeds, LS2 9JT, UK

[3]Current address: AstraZeneca, Granta Park, Cambridge, CB21 6GH, UK

```
Heavy chain

       1        10        20        30        40        50        60
IgGWFL QVQLVQSGA○EVKKPGSSVKVSCKASGGTF○○○○WFGAFTWVRQAPGQGLEWMGGIIPI○
IgGSTT ---------○--------------------○○○○ST-----------------------○

       61       70        80        90        100       110
                                                           abcddcba
IgGWFL ○FGLTNLAQNFQ○GRVTITADESTSTVYMELSSLRSEDTAVYYCARSSRIYDLNPSLTAY
IgGSTT ○--T--------○-----------------------------------------------

       113    120
IgGWFL YDMDVWGQGTMVTVSS
IgGSTT ----------------


Light chain

       1        10        20        30        40        50        60
IgGWFL QSVLTQPPS○VSAAPGQKVTISCSGSSSDI○○○○GNNYVSWYQQLPGTAPKLLIYDN○○○
IgGSTT ---------○--------------------○○○○-----------------------○○○

       61       70        80        90        100       110       120
IgGWFL ○○○○NKRPSGIP○DRFSGSK○○SGTSATLGITGLQTGDEADYYCGTWDSS○○LSAWVFGG
IgGSTT ○○○○--------○-------○○----------------------------○○--------

       121
IgGWFL GTKLTVL
IgGSTT -------
```

Supplementary Figure 1. Sequence alignment of V_H and V_L domains of IgG_{WFL} and IgG_{STT}. Dash '-' represents conserved residues. Open circle 'o' denotes IMGT numbering gaps. CDRs are highlighted in blue. Residues 35, 36 and 64 that differ between WFL and STT are shown in red.

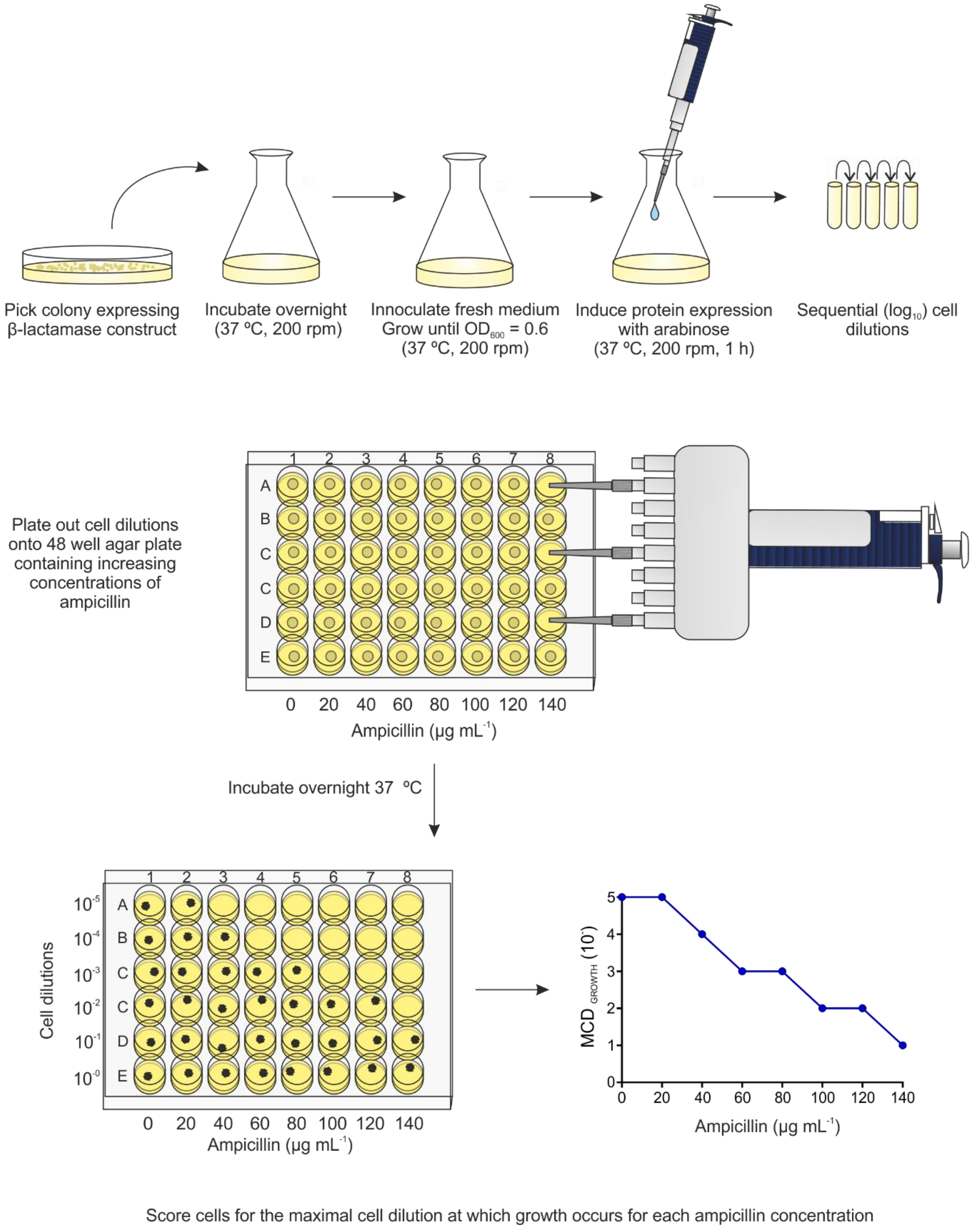

Supplementary Figure 2. Schematic of the *in vivo* growth assay. Colonies transformed with the β-lactamase fusion construct are cultured until an OD_{600} of 0.6 is reached. β-lactamase expression is induced by the addition of arabinose and cells then incubated at 37 °C for 1 h at 200 rpm. Cultures are serially diluted into 170 mM sodium chloride and 3 µL pipetted onto each well of the prepared agar plates with increasing concentrations of ampicillin in each column. Plates are incubated for 18 h at 37 °C. The maximal cell dilution at which growth occurs (MCD_{GROWTH}) is scored by visual inspection (where growth is scored as positive when > 10 individual colonies are visible on the plate).

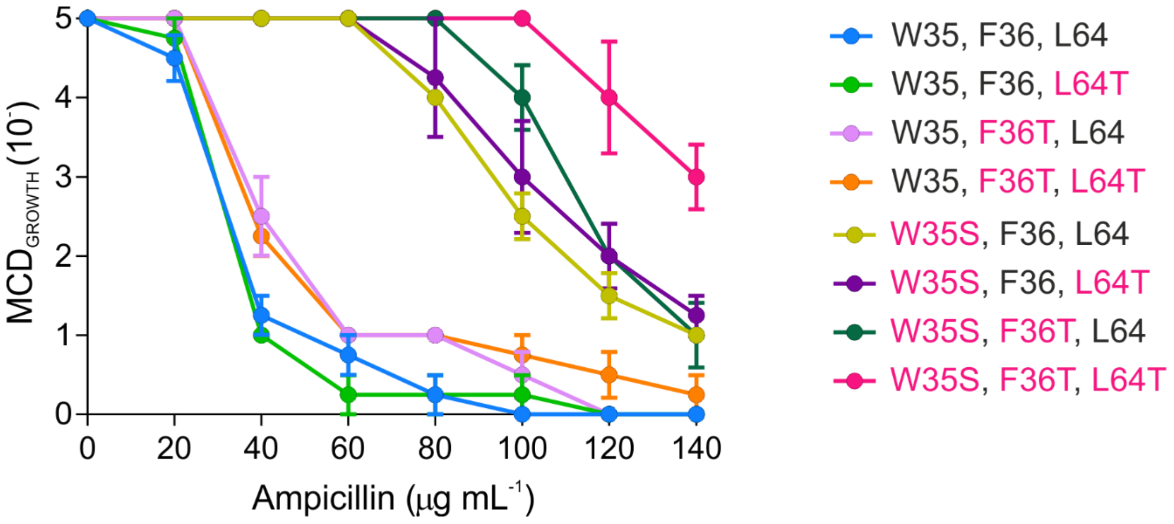

Supplementary Figure 3. *In vivo* growth curves of βLa-scFv variants containing WFL, STT, or one, two or all three amino acid substitutions between WFL and STT. Pink font indicates mutations away from WFL. Error bars represent s.e.m (n = 4 biologically independent experiments). Source data are provided as a Source Data file.

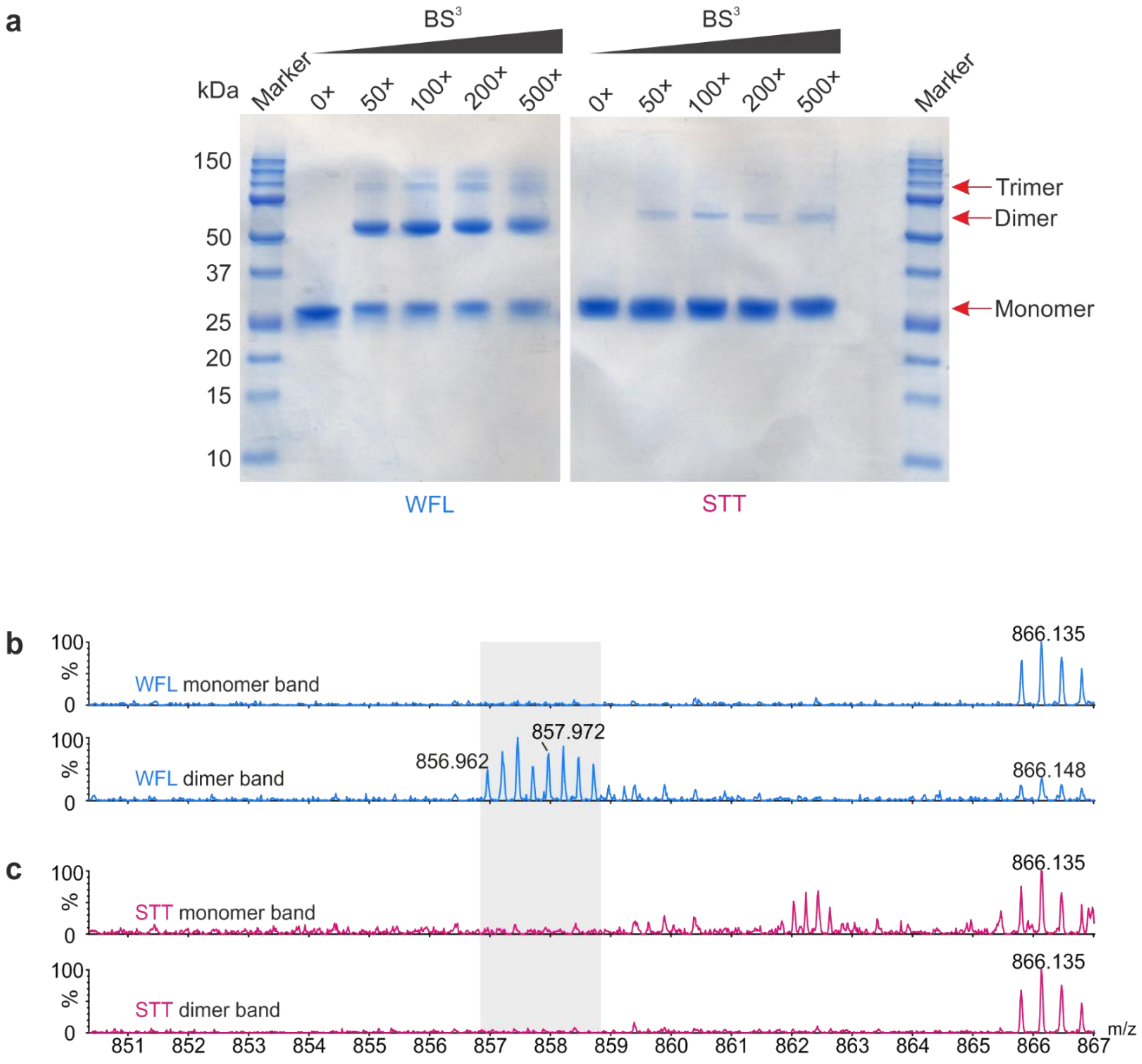

Supplementary Figure 4. Chemical cross-linking and digestion of scFvs. (**a**) Chemical cross-linking of scFvs WFL and STT as a function of BS^3 concentration (0–500-fold molar excess over [scFv]). Mass spectra of digests from the excised monomer or dimer bands of (**b**) $scFv_{WFL}$, and (**c**) $scFv_{STT}$. The cross-link unique to WFL is labelled at 856.96 m/z. Note that few, if any, peptides could be identified for $scFv_{STT}$ consistent with their reduced aggregation propensity. $scFv_{WFL}$ reveals the same, unique cross-linked peptide MQVQLVQSGAEVK (V_H) and LLIYDNNKRPSGIPDR (V_L) (**Supplementary Fig. 5**) identified in IgG_{WFL} (previously named MEDI1912)[1]. Source data are provided as a Source Data file.

Supplementary Figure 5. MS/MS sequencing data of the isolated cross-linked peptide (856.96 m/z) from the digested dimer band of cross-linked $scFv_{WFL}$ in **Supplementary Figure 4b**. The two cross-linked peptides MQVQLVQSGAEVK (V_H) and LLIYDNNKRPSGIPDR (V_L) are labelled as alpha (green) and beta (blue), respectively with the identified y-ions highlighted below.

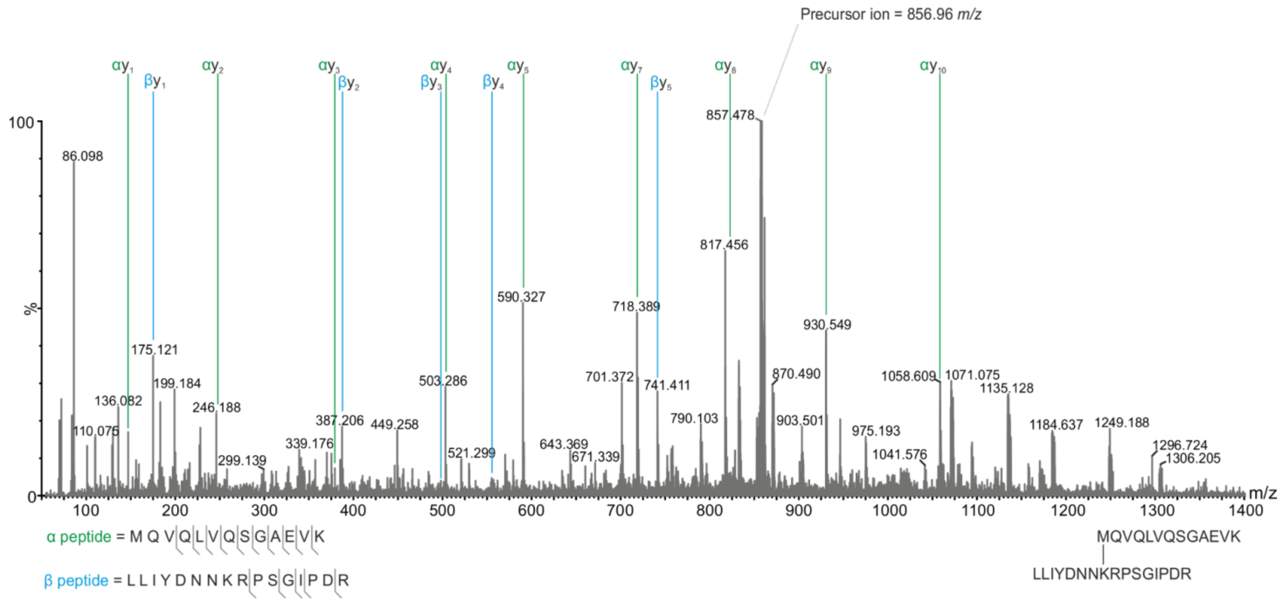

a

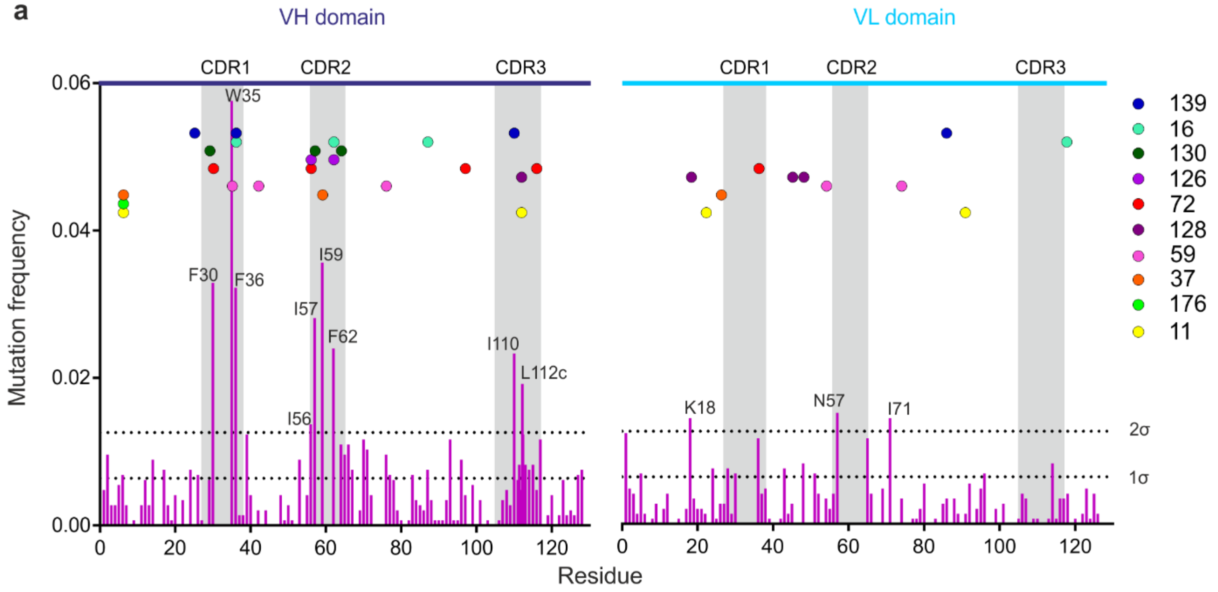

b

Variant	Mutation(s)				
139	S25G (V_H)	F36S(V_H)	I110T (V_H)	S86T (V_L)	
16	F36S (V_H)	F62S (V_H)	V87A (V_H)	F118I (V_L)	
130	T29A (V_H)	I57T (V_H)	L64H (V_H)		
126	I56F (V_H)	F62S (V_H)			
72	F30S (V_H)	I56T (V_H)	E97D (V_H)	D116G (V_H)	N36D (V_L)
128	Y112D (V_H)	K18E (V_L)	L45P (V_L)	T48A (V_L)	
59	W35R (V_H)	V42A (V_H)	V76A (V_H)	I54T (V_L)	D74G (V_L)
37	Q6P (V_H)	I59T (V_H)	S26P(V_L)		
176	Q6R (V_H)				
11	Q6R (V_H)	T112bA (V_H)	S22P (V_L)	I91V (V_L)	

Supplementary Figure 6. Location and identity of substituted residues within the ten evolved IgGs selected for biophysical characterisation compared with mutational frequency of each residue in the βLa-scFv$_{WFL}$* library. (**a**) Location of amino acid substitutions (showed by filled circles) in each of the 10 selected constructs (coloured according to the key), overlaid on the mutational frequency graph of the screened βLa-scFv$_{WFL}$* library. (**b**) Inventory of the substitutions found in the ten constructs selected for *in vitro* analysis (see **Fig. 3a**). The variant names are coloured as in (a).

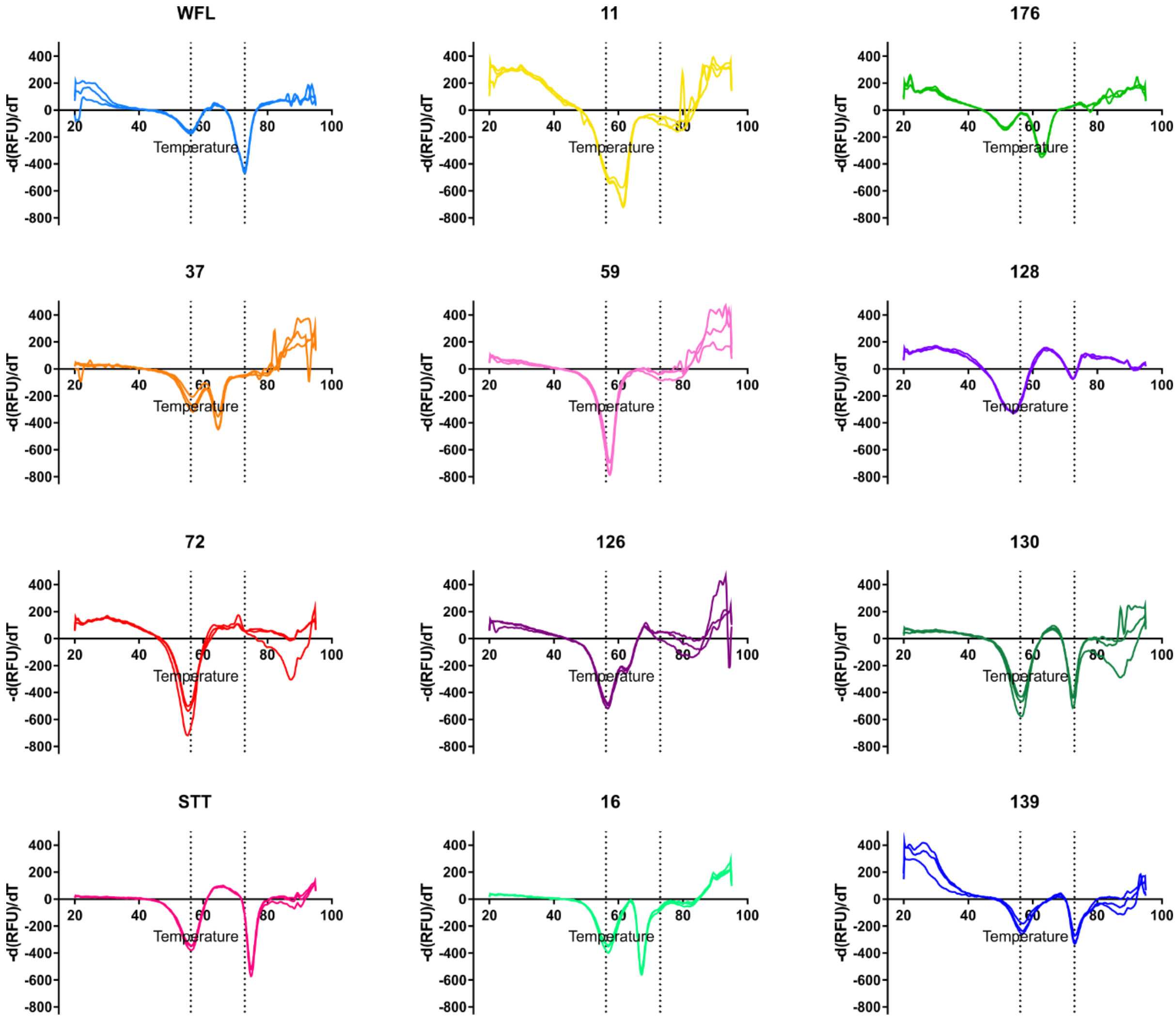

Supplementary Figure 7. Thermal stability of IgG_{WFL}, IgG_{STT} and the ten evolved variants selected for study measured by differential scanning fluorimetry (DSF). Triplicate biological repeat data are presented as first derivatives of relative fluorescence units (RFU) versus temperature (°C). Graphs are arranged by increasing *in vivo* growth assay score from top left to bottom right. The Tm values of IgG_{WFL} (56 and 73 °C) are shown on all plots as dotted lines to enable comparison of the data. Tm values are listed in **Supplementary Table 1**. Source data are provided as a Source Data file.

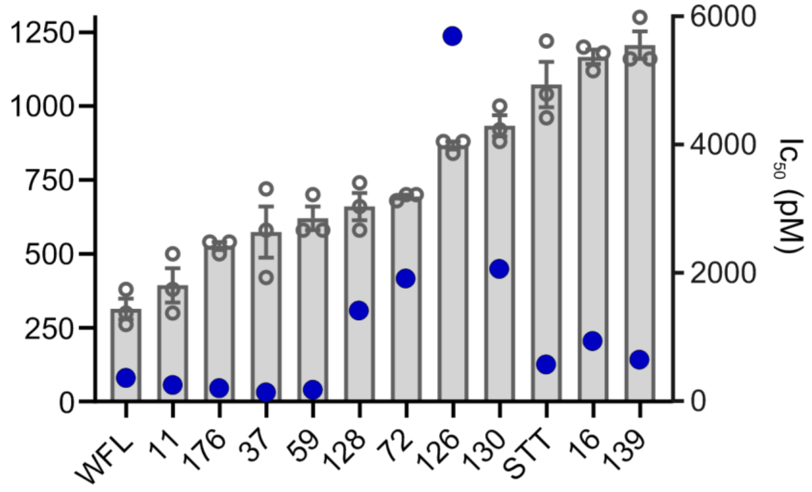

Supplementary Figure 8. *In vivo* growth scores of βla-scFv$_{WFL}$ variants (bars). Error bars indicate s.e.m (n = 4 biologically independent experiments) overlaid with IC_{50} values (blue circles). Note that the error bars for the IC_{50} values are smaller than the symbol (n = 3 technical repeats)).

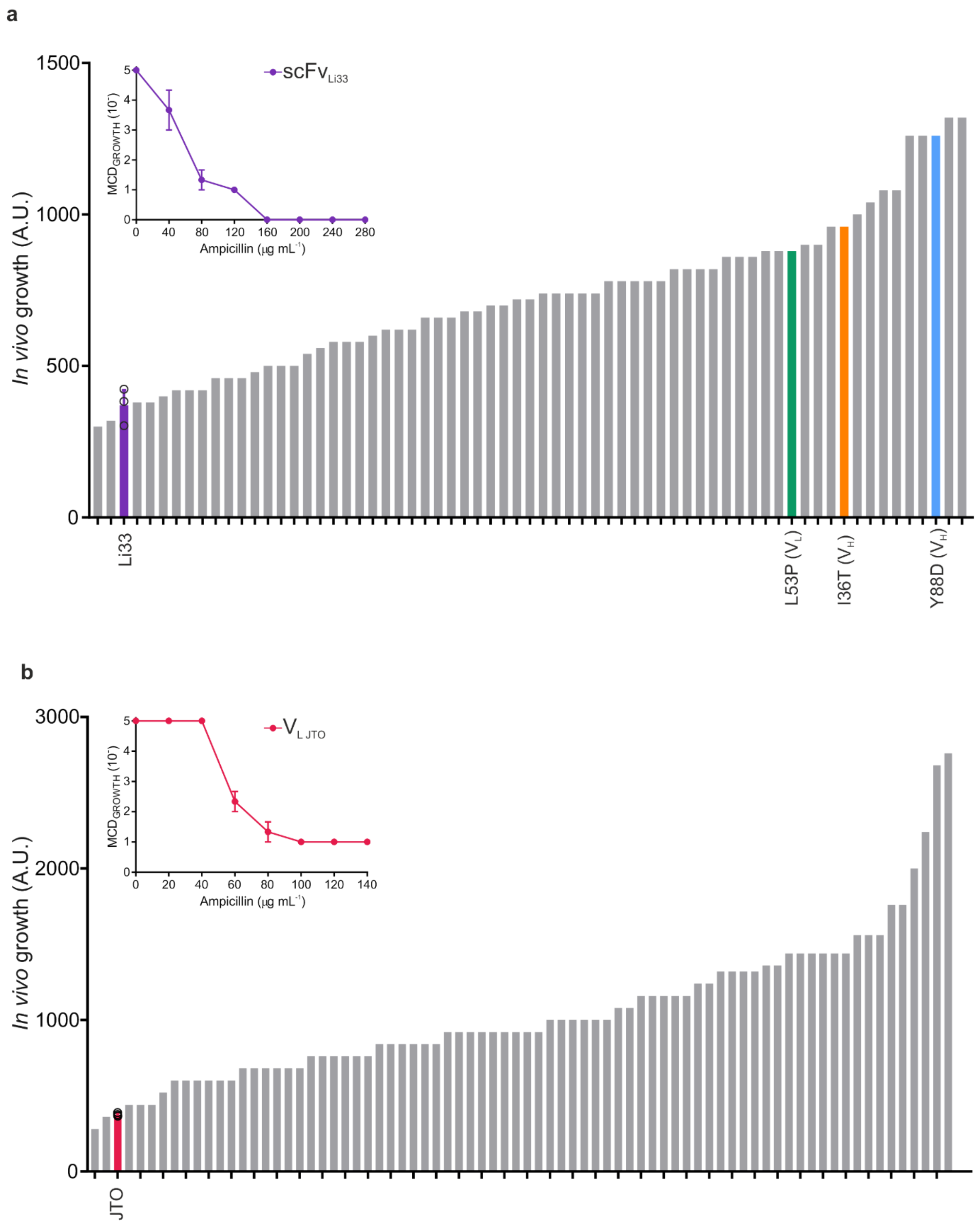

Supplementary Figure 9. *In vivo* growth scores of evolved $scFv_{Li33}$ and JTO. (**a**) *In vivo* growth scores of 66 evolved βLa-$scFv_{Li33}$ variants. Wild-type $scFv_{Li33}$ together with the frequently mutated 'hotspot' residues L53P (V_L), I36T (V_H) and the highest scoring single point mutation, Y88D (V_H) are highlighted. The full *in vivo* growth curve for wild-type βLa-$scFv_{Li33}$ is shown inset. Error bars = s.e.m (n = 3 biologically independent experiments). (**b**) *In vivo* growth scores of 75 evolved βLa-JTO variants (JTO highlighted). The full *in vivo* growth curve for wild-type βLa-JTO is shown inset. Initial selection for both constructs was performed at 140 μg/mL ampicillin. Error bars = s.e.m (n = 3 biologically independent experiments). Source data are provided as a Source Data file.

Heavy chain

```
          1        10        20        30        40        50        60
IgG_WFL   QVQLVQSGA○EVKKPGSSVKVSCKASGGTF○○○○WFGAFTWVRQAPGQGLEWMGGIIPI○
IgG_Li33  E---LE--G○GLVQ--G-LRL--A---F--○○○○SIYPMF-------K----VSW-G-S○

          61       70        80        90        100       110 abcddcba
IgG_WFL   ○FGLTNLAQNFQ○GRVTITADESTSTVYMELSSLRSEDTAVYYCARSSRIYDLNPSLTAY
IgG_Li33  ○G-I-KY-DSVK○--F--SR-N-KN-L-LQMN---A----T-----EGHN○○○○○○○○○D

          113    120
IgG_WFL   YDMDVWGQGTMVTVSS
IgG_Li33  WYF-L--R--L-----
```

Light chain

```
          1        10        20        30        40        50        60
IgG_WFL   QSVLTQPPS○VSAAPGQKVTISCSGSSSDI○○○○GNNYVSWYQQLPGTAPKLLIYDN○○○
IgG_Li33  DIQM--S-GTL-LS--ERA-L--RA-Q-V○○○○○○SS-LA----K--Q--R-----A○○○

          61       70        80        90        100       110       120
IgG_WFL   ○○○○NKRPSGIP○DRFSGSK○○SGTSATLGITGLQTGDEADYYCGTWDSS○○LSAWVFGG
IgG_Li33  ○○○○SN-AT---○A-----G○○---EF--T-SS--SE-F-V---QQYDK○○○○VPLT---

          121
IgG_WFL   GTKLTVL
IgG_Li33  ---VEIK
```

Supplementary Figure 10. Sequence alignment of V_H and V_L domains of IgG_{WFL} and IgG_{Li33}. Dash '-' represents identical residues. Open circle 'o' denotes IMGT numbering gaps. CDRs are highlighted in blue.

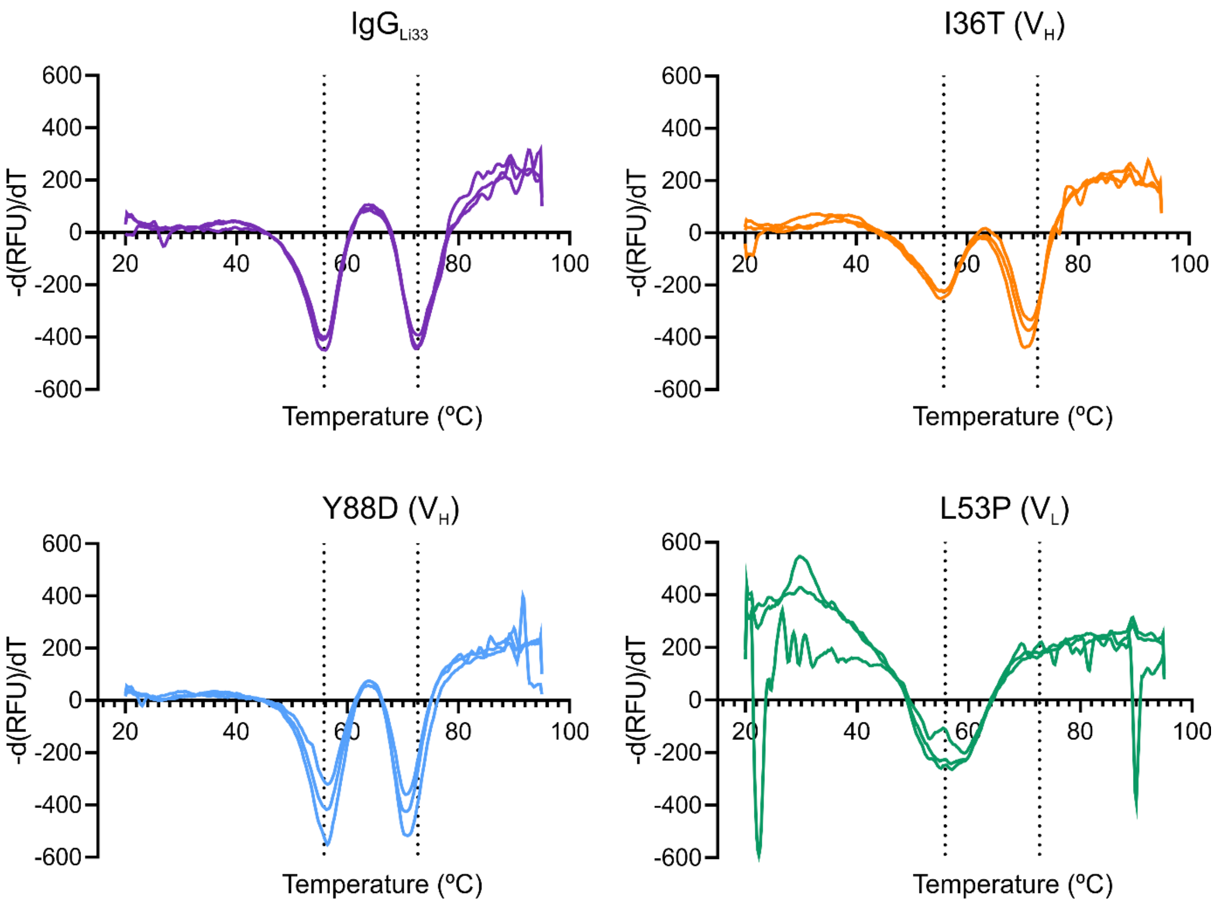

Supplementary Figure 11. Thermal stability of IgG$_{Li33}$ and the three evolved variants selected for study measured by DSF. Triplicate biological repeat data are presented as first derivatives of relative fluorescence units (RFU) versus temperature (°C). Graphs are arranged by increasing *in vivo* growth assay score from top left to bottom right. The thermal unfolding transitions of IgG$_{Li33}$ (56 and 73 °C) are shown on all plots as dotted lines to enable comparison of the data. Tm values are listed in **Supplementary Table 1**. Source data are provided as a Source Data file.

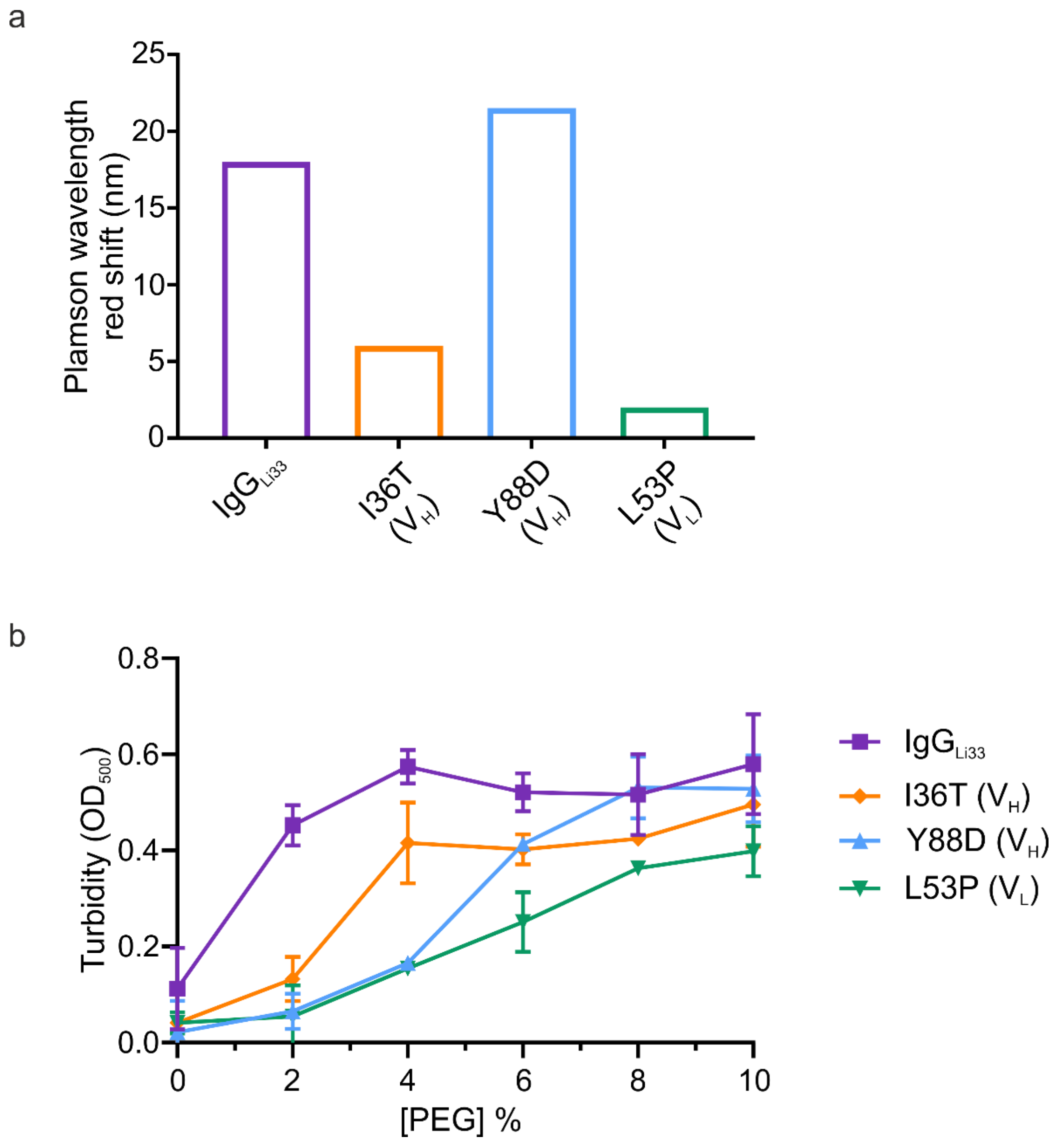

Supplementary Figure 12. Biophysical assessment of IgG$_{Li33}$ evolved variants (**a**) AC-SINS data of IgG$_{Li33}$ show that two out of three evolved IgGs have reduced self-association propensity compared with wild-type IgG$_{Li33}$. (**b**) Polyethylene glycol (PEG) precipitation of IgG$_{Li33}$ and three evolved variants quantified by turbidity at 500 nm showing that the evolved IgGs have increased solubility compared with wild-type IgG$_{Li33}$. Error bars = SD (n = 3 technical repeats). Source data are provided as a Source Data file.

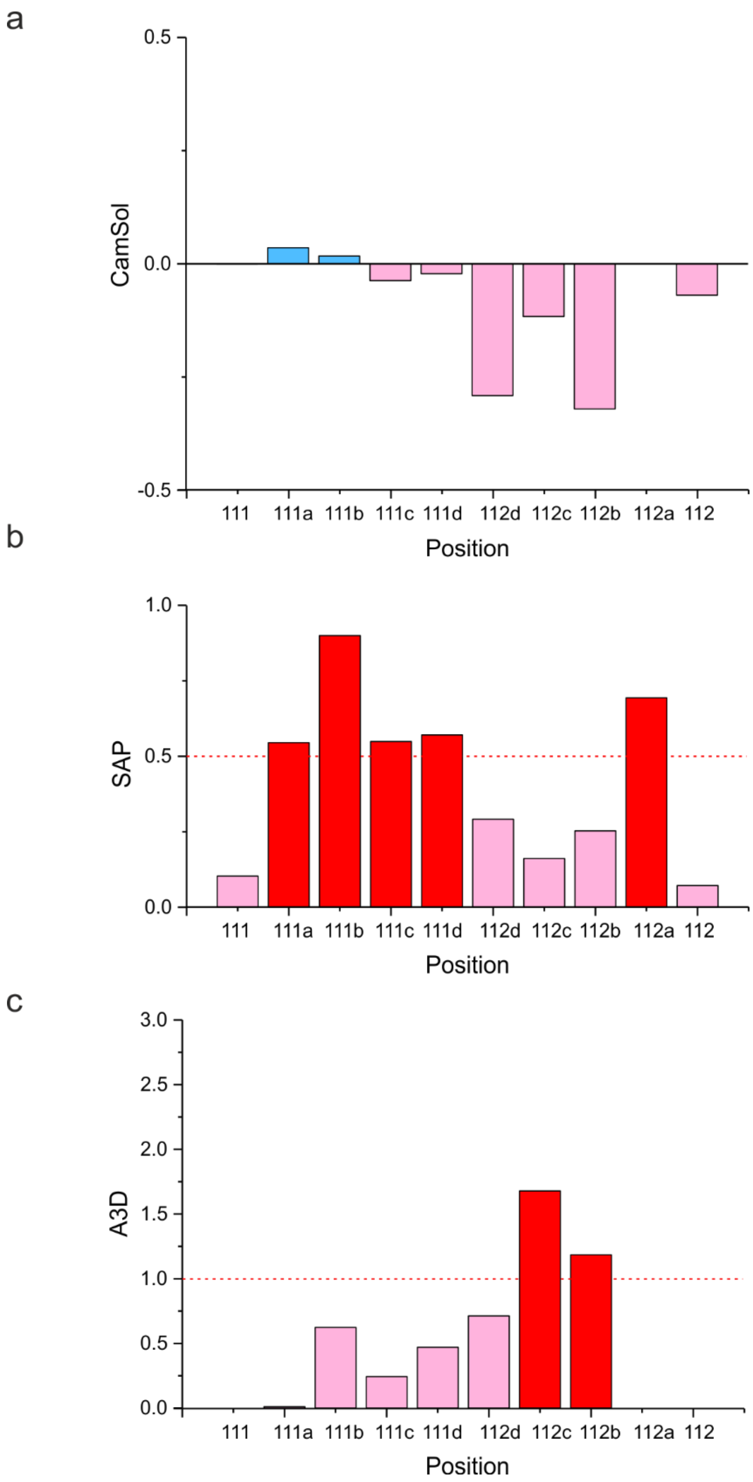

Supplementary Figure 13. Data from **Fig. 5b** showing an expanded view of residues 111-112 in CDR3 (V_H), IMGT numbering. Source data are provided as a Source Data file.

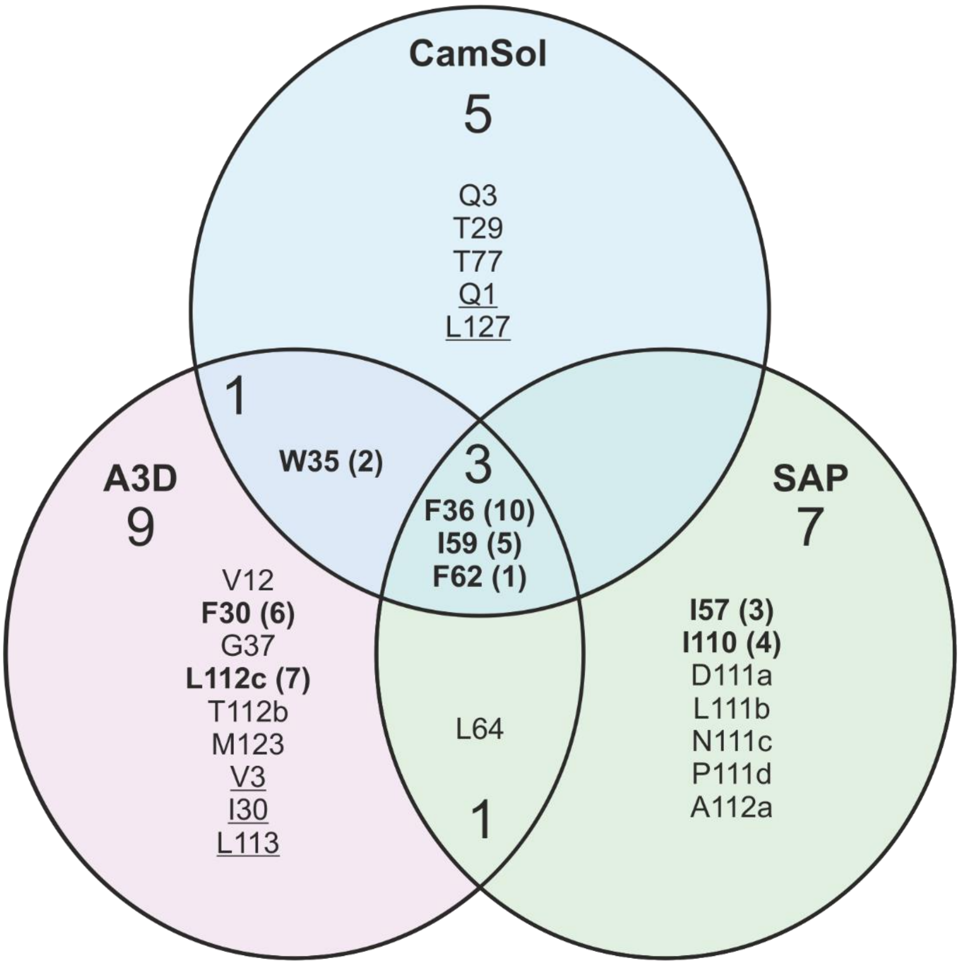

Supplementary Figure 14. Comparison of aggregation prone/insoluble residues identified by Camsol, Aggrescan3D and SAP. Residues were identified as aggregation prone/insoluble from CamSol (output score < -1), Aggrescan3D (> 1) or SAP (> 0.5). V_L domain residues are underlined. Residues in bold are those identified as hotspots using the TPBLA. The number in brackets corresponds to the rank in aggregation propensity based on the *in vivo* growth score when introduced as single substitutions (see **Supplementary Fig. 15**).

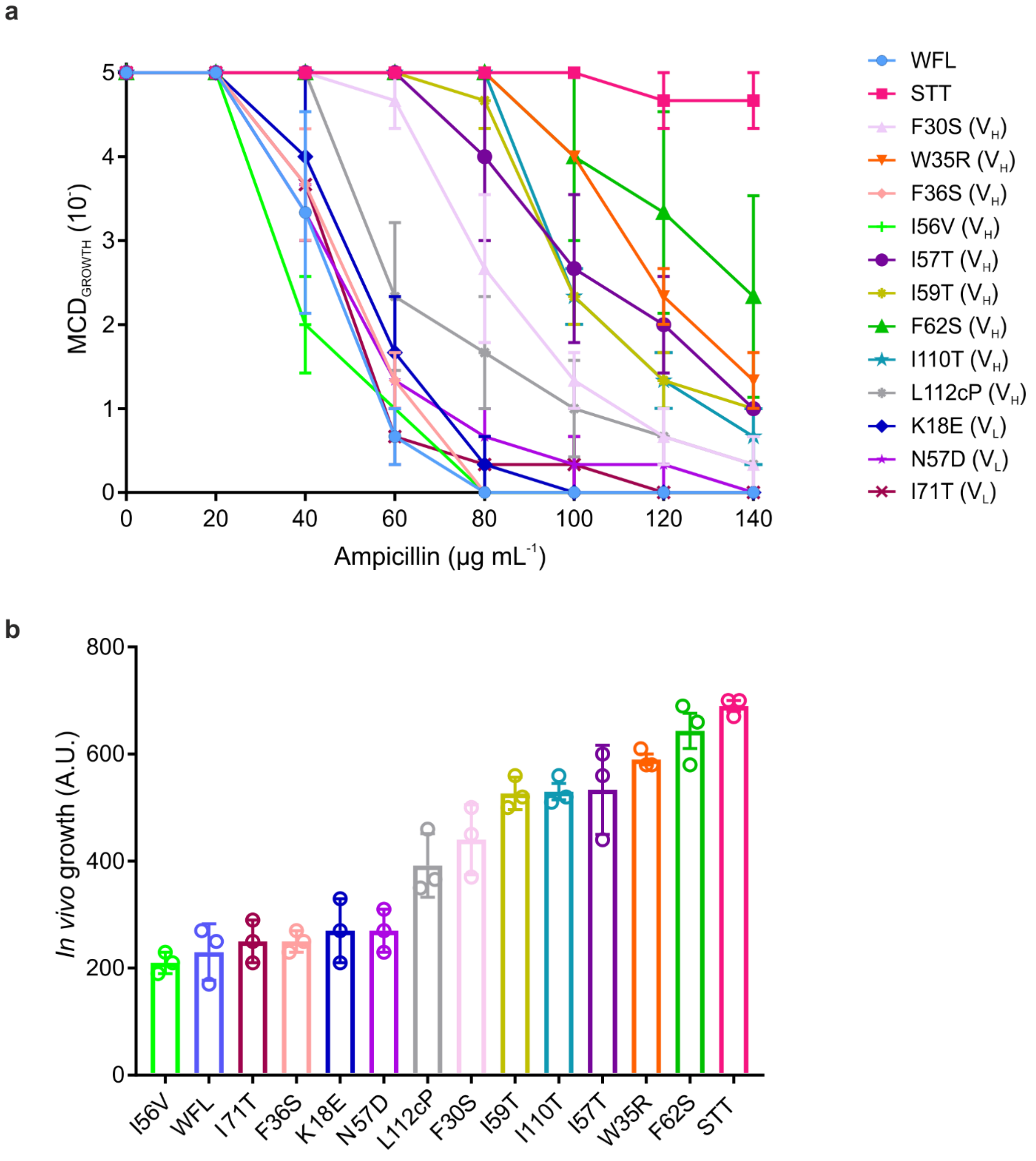

Supplementary Figure 15. *In vivo* growth assay of the twelve hotspot residues in βLa-scFv$_{WFL}$ containing the most commonly substituted amino-acid. (**a**) Survival curves of each variant. (**b**) Area under the curve scores for each variant calculated from the data in (**a**). Error bars indicate the standard error of the mean (n = 3 biologically independent experiments). Source data are provided as a Source Data file.

Supplementary Tables

IgG		Tm1 (°C)	Tm2 (°C)
**WFL**	WT (WFL)	56.1 ± 0.1	72.9 ± 0.1
	11	57.4 ± 0.0	61.3 ± 0.1
	176	51.5 ± 0.2	62.8 ± 0.0
	37	56.7 ± 0.2	64.6 ± 0.0
	59	57.3 ± 0.1	*
	128	54.1 ± 0.1	72.4 ± 0.0
	72	55.2 ± 0.0	*
	126	56.7 ± 0.1	62.1 ± 0.1
	130	56.4 ± 0.2	72.4 ± 0.0
	STT	56.3 ± 0.1	74.8 ± 0.0
	16	56.8 ± 0.0	67.2 ± 0.0
	139	56.8 ± 0.0	73 ± 0.0
**Li33**	WT (Li33)	55.8 ± 0.4	72.7 ± 0.8
	Y88D	56.2 ± 0.2	71.2 ± 0.9
	I36T	55.8 ± 0.4	71.0 ± 0.7
	L53P	55.5 ± 1.1	*

Supplementary Table 1: Thermal stabilities of IgG_{WFL}, IgG_{Li33} and their variants showing transition mid-point temperatures (Tm1 and Tm2) from first and second peaks of first derivative DSF measurements. Errors represent SD (n = 3 biological repeats). * = only one transition temperature detected. Thermal unfolding profiles for each variant are shown **in Supplementary Figures 7 and 11** for IgG_{WFL} and IgG_{Li33}, respectively. Source data are provided as a Source Data file.

**Evolved construct (IgG)**	**IC_{50} (pM)**
11	251 ± 4.8
176	201 ± 6.4
37	136 ± 3.1
59	177 ± 5.9
128	1413 ± 4.2
72	1911 ± 2.0
126	5693 ± 4.5
130	2063 ± 3.2
16	935 ± 2.6
139	649 ± 6.9
WFL	363 ± 2.4
STT	573 ± 4.0
MEDI578	36760 ± 2.8

Supplementary Table 2: IC_{50} values of IgG_{WFL}, IgG_{STT} and the ten selected evolved variants binding to nerve growth factor (NGF) measured using an epitope competition assay (n = 3 technical repeats, error = s.e.m). IgG MEDI578 is the parent antibody subjected to affinity panning which generated IgG_{WFL}. Source data are provided as a Source Data file.

Residue	Computational tool		
	CamSol	SAP	Aggrescan3D
F30	-0.203	-0.107	2.30
W35	-1.65	0.490	2.23
F36	-2.41	0.745	2.87
I56	-0.074	0.165	0.641
I57	0	0.192	0
I59	-1.50	0.71	0.64
F62	-1.087	1.10	2.52
I110	-0.053	0.56	0.722
L112c	-0.116	0.161	1.67
K18	-0.046	-0.429	-2.28
N57	-1.98	-0.480	0.236
I71	0.382	-0.419	0.299

Supplementary Table 3: Analysis of hotspot residues using CamSol, SAP and Aggrescan3D. The numerical score is colour coded based on arbitrary cut-off values for each algorithm in line with **Fig. 5**: Structurally corrected CamSol, +1 indicates soluble and -1 indicates insoluble; SAP (using a 10 Å radius) where values > 0.5 and -0.5 are significant; and Aggrescan3D, where values > 1 and <-1 are significant. Source data are provided as a Source Data file.

**DNA**	ATGAGTATTCAACATTTCCGTGTCGCCCTTATTCCCTTTTTTGCGGCATTTTGCCTTCCTGTTTTTGC<br>TCACCCAGAAACGCTGGTGAAAGTAAAAGATGCTGAAGATCAGTTGGGTGCACGAGTGGGTTAC<br>ATCGAACTGGATCTCAACAGCGGTAAGATCCTTGAGAGTTTTCGCCCCGAAGAACGTTTTCCAAT<br>GATGAGCACTTTTAAAGTTCTGCTATGTGGCGCGGTATTATCCCGTGTTGACGCCGGGCAAGAGC<br>AACTCGGTCGCCGCATACACTATTCTCAGAATGACTTGGTTGAGTACTCACCAGTCACAGAAAAG<br>CATCTTACGGATGGCATGACAGTAAGAGAATTATGCAGTGCTGCCATAACCATGAGTGATAACAC<br>TGCGGCCAACTTACTTCTGACAACGATCGGAGGACCGAAGGAGCTAACCGCTTTTTTGCACAACA<br>TGGGGGATCATGTAACTCGCCTTGATCGTTGGGAACCGGAGCTGAATGAAGCCATACCAAACGA<br>CGAGCGTGACACCACGATGCCTGCAGCAATGGCAACAACGTTGCGCAAACTATTAACTGGCGAA<br>CTA**GGTGGTGGTGGTTCTGGTGGTGGTGG**CTCGAGCTCAGGATCC**GGGAGCGGTTCCGGAAGC**<br>**GGAGGAGGTGGTTCAGGCGGAGGTGGAAGC**TTGACTCTAGCTAGCCGGCAGCAGCTCATAGAC<br>TGGATGGAGGCGGATAAAGTTGCAGGACCACTTCTGCGCTCGGCCCTTCCGGCTGGCTGGTTTA<br>TTGCTGATAAATCTGGAGCCGGTGAGCGTGGGTCTCGCGGTATCATTGCAGCACTGGGGCCAGA<br>TGGTAAGCCCTCCCGTATCGTAGTTATCTACACGACGGGGAGTCAGGCAACTATGGATGAACGA<br>AATAGACAGATCGCTGAGATAGGTGCCTCACTGATTAAGCATTGGTAA
**Amino Acid**	MSIQHFRVALIPFFAAFCLPVFAHPETLVKVKDAEDQLGARVGYIELDLNSGKILESFRPEERFPMMST<br>FKVLLCGAVLSRVDAGQEQLGRRIHYSQNDLVEYSPVTEKHLTDGMTVRELCSAAITMSDNTAANLLL<br>TTIGGPKELTAFLHNMGDHVTRLDRWEPELNEAIPNDERDTTMPAAMATTLRKLLTGEL**GGGGSGG**<br>**GGSSSGSGSGSGSGGGGSGGGGS**LTLASRQQLIDWMEADKVAGPLLRSALPAGWFIADKSGAGER<br>GSRGIIAALGPDGKPSRIVVIYTTGSQATMDERNRQIAEIGASLIKHW

Supplementary Table 4. DNA and amino acid sequences of the β-lactamase-linker construct. The periplasmic signal sequence is in purple. The G/S-rich linker is shown in bold. The restriction sites XhoI and BamHI are shown in blue and green, respectively. The start and stop codons are underlined.

Construct	Amino Acid Sequence
scFv WFL	QVQLVQSGAEVKKPGSSVKVSCKASGGTFWFGAFTWVRQAPGQGLEWMGGIIPIFGLTNLAQNF QGRVTITADESTSTVYMELSSLRSEDTAVYYCARSSRIYDLNPSLTAYYDMDVWGQGTMVTVSSGG GSSGGGGSGGGGGAQSVLTQPPSVSAAPGQKVTISCSGSSSDIGNNYVSWYQQLPGTAPKLLIYD NNKRPSGIPDRFSGSKSGTSATLGITGLQTGDEADYYCGTWDSSLSAWVFGGGTKLTVL
scFv STT	QVQLVQSGAEVKKPGSSVKVSCKASGGTFSTGAFTWVRQAPGQGLEWMGGIIPIFGLTNLAQNF QGRVTITADESTSTVYMELSSLRSEDTAVYYCARSSRIYDLNPSLTAYYDMDVWGQGTMVTVSSGG GSSGGGGSGGGGGAQSVLTQPPSVSAAPGQKVTISCSGSSSDIGNNYVSWYQQLPGTAPKLLIYD NNKRPSGIPDRFSGSKSGTSATLGITGLQTGDEADYYCGTWDSSLSAWVFGGGTKLTVL
GCSF	MTPLGPASSLPQSFLLKCLEQVRKIQGDGAALQEKLCATYKLCHPEELVLLGHSLGIPWAPLSSCPSQ ALQLAGCLSQLHSGLFLYQGLLQALEGISPELGPTLDTLQLDVADFATTIWQQMEELGMAPALQPT QGAMPAFASAFQRRAGGVLVASHLQSFLEVSYRVLRHLAQP
GCSF-C3	MTPLGPASSLPQSFLLKGLEQVRKIQGDGAALQEKLCATYKLCHPEELVLLGHSLGIPRAPLSSCPSQ ALRLAGCLSQLHSGLLLYQGLLQALEGISPELGPTLDTLQLDVADFATTIWQQMEELGMAPALQPT QGAMPAFASAFQRRAGGVLVASHLQSFLEVSYRVLRHLAQP
Dp47d	EVQLLESGGGLVQPGGSLRLSCAASGFTFSSYAMSWVRQAPGKGLEWVSAISGSGGSTYYADSVK GRFTISRDNSKNTLYLQMNSLRAEDTAVYYCAKSYGAFDYWGQGTLVTVSS
HEL4	EVQLLESGGGLVQPGGSLRLSCAASGFRISDEDMGWVRQAPGKGLEWVSSIYGPSGSTYYADSVK GRFTISRDNSKNTLYLQMNSLRAEDTAVYYCASALEPLSEPLGFWGQGTLVTVSS
scFv Li33	EVQLLESGGGLVQPGGSLRLSCAASGFTFSIYPMFWVRQAPGKGLEWVSWIGPSGGITKYADSVK GRFTISRDNSKNTLYLQMNSLRAEDTATYYCAREGHNDWYFDLWGRGTLVTVSSGGGGSGGGGS GGGGSGGGGSDIQMTQSPGTLSLSPGERATLSCRASQSVSSYLAWYQQKPGQAPRLLIYDASNRA TGIPARFSGSGSGTEFTLTISSLQSEDFAVYYCQQYDKWPLTFGGGTKVEIK
V_L JTO	NFMLNQPHSVSESPGKTVTISCTRSSGNIDSNYVQWYQQRPGSAPITVIYEDNQRPSGVPDRFAGS IDRSSNSASLTISGLKTEDEADYYCQSYDARNVVFGGGTRLTVL

Supplementary Table 5. Amino acid sequences of test proteins and their variants. Sequence differences between variant pairs are highlighted in yellow.

**DNA**	ATGAGTATTCAACATTTCCGTGTCGCCCTTATTCCCTTTTTTGCGGCATTTTGCCTTCCTGTTTTTGC<br>TCACCCAGAAACGCTGGTGAAAGTAAAAGATGCTGAAGATCAGTTGGGTGCACGAGTGGGTTAC<br>ATCGAACTGGATCTCAACAGCGGTAAGATCCTTGAGAGTTTTCGCCCCGAAGAACGTTTTCCAAT<br>GATGAGCACTTTTAAAGTTCTGCTATGTGGCGCGGTATTATCCCGTGTTGACGCCGGGCAAGAGC<br>AACTCGGTCGCCGCATACACTATTCTCAGAATGACTTGGTTGAGTACTCACCAGTCACAGAAAAG<br>CATCTTACGGATGGCATGACAGTAAGAGAATTATGCAGTGCTGCCATAACCATGAGTGATAACAC<br>TGCGGCCAACTTACTTCTGACAACGATCGGAGGACCGAAGGAGCTAACCGCTTTTTTGCACAACA<br>TGGGGGATCATGTAACTCGCCTTGATCGTTGGGAACCGGAGCTGAATGAAGCCATACCAAACGA<br>CGAGCGTGACACCACGATGCCTGCAGCAATGGCAACAACGTTGCGCAAACTATTAACTGGCGAA<br>CTA**GGTGGTGGTGGTTCTGGTGGTGGTGG**CTCGAGCCAGGTTCAGCTTGTGCAGAGCGGT<br>GCGGAGGTCAAAAAACCCGGCAGCTCTGTAAAAGTTAGCTGCAAAGCGAGTGGCGGT<br>ACGTTTTGGTTTGGGGCCTTTACTTGGGTTCGTCAAGCGCCGGGCCAGGGCTTGGAATG<br>GATGGGTGGCATTATCCCTATTTTCGGCCTCACAAACCTGGCGCAAAACTTTCAAGGTC<br>GCGTTACCATTACGGCGGACGAAAGCACCAGTACCGTCTATATGGAGCTGTCAAGCCT<br>GCGCTCAGAAGACACCGCAGTTTAATAATGTGCGCGTAGCAGCCGCATTTACGACTTGA<br>ATCCTAGCCTCACAGCGTACTACGACATGGATGTGTGGGGGCAGGGCACCATGGTTAC<br>GGTGTCGAGTGGTGGTGGGAGCAGTGGTGGAGGTGGGTCCGGGGGCGGCGGCGGCG<br>CGCAAAGCGTATTAACTCAGCCGCCGAGCGTGAGCGCAGCCCCTGGGCAGAAAGTCAC<br>CATTTCATGCAGCGGCTCCTCCAGCGATATCGGCAACAATTACGTGTCCTGGTATCAGC<br>AGCTGCCTGGCACTGCGCCGAAGCTGTTGATTTATGACAACAATAAGCGTCCCTCGGGT<br>ATTCCAGATCGTTTTTCTGGCTCTAAAAGCGGGACATCAGCGACACTGGGCATCACCGG<br>GCTGCAGACGGGGGATGAAGCCGATTATTACTGCGGGACCTGGGATAGTTCCCTGAGC<br>GCGTGGGTGTTTGGCGGGGGCACCAAACTCACCGTGCTGGGATCC**GGGAGCGGTTCCG**<br>**GAAGCGGAGGAGGTGGTTCAGGCGGAGGTGGAAGC**TTGACTCTAGCTAGCCGGCAGCAGCTC<br>ATAGACTGGATGGAGGCGGATAAAGTTGCAGGACCACTTCTGCGCTCGGCCCTTCCGGCTGGCT<br>GGTTTATTGCTGATAAATCTGGAGCCGGTGAGCGTGGGTCTCGCGGTATCATTGCAGCACTGGG<br>GCCAGATGGTAAGCCCTCCCGTATCGTAGTTATCTACACGACGGGGAGTCAGGCAACTATGGAT<br>GAACGAAATAGACAGATCGCTGAGATAGGTGCCTCACTGATTAAGCATTGGTAA

Supplementary Table 6. DNA sequence of the β-lactamase stop template. The periplasmic signal sequence is in purple. The G/S-rich linker is shown in bold. The restriction sites XhoI and BamHI are shown in blue and green, respectively. The start and stop codons are underlined. The position of the inserted stop codons are highlighted in red.

Supplementary References

1. Dobson, C. L. *et al.* Engineering the surface properties of a human monoclonal antibody prevents self-association and rapid clearance in vivo. *Sci. Rep.* **6**, 38644 (2016).
